# Long noncoding RNA NEAT1 promotes ferroptosis by modulating the miR-362-3p/MIOX axis as a ceRNA

**DOI:** 10.1038/s41418-022-00970-9

**Published:** 2022-03-25

**Authors:** Ying Zhang, Meiying Luo, Xiaohong Cui, Douglas O’Connell, Yongfei Yang

**Affiliations:** 1 Key Laboratory of Molecular Medicine and Biotherapy, School of Life Science, Beijing Institute of Technology, Beijing, 100081 China; 2 Psychiatry Department, Shanxi Bethune Hospital, Taiyuan, Shanxi 030000 China; 3 College of Medicine, Touro University, Vallejo, CA 94592 USA

**Keywords:** Cell biology, Cancer

## Abstract

Ferroptosis, a novel form of regulated cell death induced by iron-dependent lipid peroxidation, plays an essential role in the development and drug resistance of tumors. Long noncoding RNA (lncRNA) nuclear paraspeckle assembly transcript 1 (NEAT1) has been reported to be involved in the regulation of cell cycle, proliferation, apoptosis, and migration of tumor cells. However, the function and molecular mechanism of NEAT1 in regulating ferroptosis in tumors remain unclear. Here, we found that ferroptosis inducers erastin and RSL3 increased NEAT1 expression by promoting the binding of p53 to the NEAT1 promoter. Induced NEAT1 promoted the expression of MIOX by competitively binding to miR-362-3p. MIOX increased ROS production and decreased the intracellular levels of NADPH and GSH, resulting in enhanced erastin- and RSL3-induced ferroptosis. Importantly, overexpression of NEAT1 increased the anti-tumor activity of erastin and RSL3 by enhancing ferroptosis both in vitro and in vivo. Collectively, these data suggest that NEAT1 plays a novel and indispensable role in ferroptosis by regulating miR-362-3p and MIOX. Considering the clinical findings that HCC patients are insensitive to chemotherapy and immunotherapy, ferroptosis induction may be a promising therapeutic strategy for HCC patients with high NEAT1 expression.

## Introduction

Ferroptosis is a recently discovered unique cell death process with excessive accumulation of iron-dependent lipoperoxides, and different from other forms of regulated cell death in terms of morphology, genetics, and biochemistry [1]. Intracellularly, iron reacts with hydrogen peroxide as a pro-oxidant to produce lipid reactive oxygen species (ROS) [2], which are usually converted to nontoxic lipid alcohols by glutathione peroxidase 4 (GPX4) and glutathione (GSH) [3]. Erastin and RSL3 are two small-molecule compounds that induce ferroptosis in cancer cells. Erastin inhibits system X_c_^-^ mediated cystine uptake, leading to GSH depletion and increased lipid ROS, while RSL3 directly binds to the GPX4 activation domain, inactivating GPX4 and inducing ferroptosis [4].

LncRNAs are a class of RNAs with more than 200 nucleotides in length [5]. Although lncRNAs do not have protein-coding capabilities, they play essential roles in various biological processes and diseases [6]. Aberrant regulation of lncRNAs is associated with tumorigenesis, metastasis, and drug resistance [7, 8]. LncRNA can mechanically act as a competing endogenous RNA (ceRNA), and ceRNA competitively binds with miRNAs to reduce miRNA regulation on their target mRNAs [9]. Recent studies have identified several lncRNAs involved in ferroptosis. PVT1 locus regulates ferroptosis by regulating TP53 and TFR1 through miR-214 in brain ischemia/reperfusion [10], LINC00336 as ceRNA inhibits miR-6852-promoted ferroptosis in lung cancer [11]. However, the regulatory mechanism of lncRNAs in ferroptosis is still not fully understood.

NEAT1 is an important lncRNA that has two transcripts NEAT1_1 (3.7 kb) and NEAT1_2 (23 kb), and is highly expressed in a variety of human tumors [12]. Overexpression of NEAT1 promotes tumor development and is associated with poor prognosis in tumor patients [13]. Mechanistically, NEAT1 acts as a sponge to absorb miRNA, thereby promoting the growth of tumor cells. For example, NEAT1, stabilized by LIN28, acts as a ceRNA for miR-506 to promote the progression of high-grade serous ovarian cancer [14]. In breast cancer, NEAT1 regulates ZEB1 through competitive binding with miR-448 to induce cell proliferation, migration, and invasion [15]. However, the role of NEAT1 in ferroptosis and its molecular mechanisms remains uninvestigated.

Myo-inositol oxygenase (MIOX) is a 33 kDa non-heme ferritin, which metabolizes inositol to D-glucuronic acid via the glucuronic acid-xylose pathway [16]. The transcription of MIOX is regulated by oxidative stress, free fatty acids, and a high-glucose environment [17]. Upregulation of MIOX promotes ROS production and reduced nicotinamide adenine dinucleotide phosphate (NADPH) and GSH, resulting in decreased cellular antioxidant capacity [18]. Here, we found that NEAT1 is upregulated in HCC cells after erastin- and RSL3-treatment. Highly expressed NEAT1 acted as a ceRNA to attract miR-362-3p to increase the expression of MIOX indirectly, thereby promoting erastin- and RSL3-induced ferroptosis in HCC cells. Our findings outline the critical role of NEAT1 in ferroptosis and its regulatory mechanism, suggesting that ferroptosis induction may be a promising therapeutic strategy for tumor patients with high expression levels of NEAT1.

## Materials and methods

### Cell lines and culture conditions

HEK293T (ATCC^®^CRL-3216) and HepG2 (ATCC^®^HB-8065) cell lines were obtained from the American Type Culture Collection (ATCC) (Manassas, VA, USA). HuH-7 (JCRB0403) was obtained from the Japanese Collection of Research Bioresources cell bank (Tokyo, Japan). All cells were cultured in Dulbecco’s modified Eagle’s medium containing 10% fetal bovine serum, 1% penicillin–streptomycin (Gibco-BRL), and 2 mM L-glutamine at 37 °C with 5% CO_2_. Transfections were performed using RNAiMax transfection reagent (Invitrogen) or Lipofectamine 2000 (Invitrogen) according to the manufacturer’s instructions. All cells were confirmed to be free of mycoplasma contamination by using cell culture contamination detection kit (Thermo). D-glucaric acid (21236) was obtained from Sigma (St. Louis, MO).

### Antibody and chemicals

The following antibodies were used in this study for western blot (WB) analysis and immunohistochemistry (IHC): MIOX (ab154639, Abcam, 1:1000 for WB, 1:100 for IHC), Actin (PA116889, Thermo, 1:1000 for WB), 4-HNE (ab46545, Abcam, 1:100 for IHC). Horseradish peroxidase (HRP)-labeled secondary antibody conjugates were purchased from Thermo. RSL3 (#S8155) and erastin (#E7781) were obtained from Selleck Chemicals (Houston, TX, USA).

### Plasmids

To generate miR-362-3p overexpression constructs, a 306 bp fragment upstream and downstream of the pre-miR-362-3p was amplified from HEK293T cDNA by PCR (forward primer, 5′-TGCCTTTCTGTAAAGTCC-3′ and reverse primer, 5′-CGTTGTATCTCCACCACC-3′) and cloned into pcDNA5/FRT/TO vector with KpnI and XhoI sites. The anti-scramble oligos (AM17010) and anti-miR-362-3p (MH12485) were obtained from Thermo. The full-length cDNA of human NEAT1 and MIOX were cloned from HEK293T cDNA by PCR. The NEAT1, MIOX, and NEAT1-MUT were constructed by cloning the cDNA of the full-length or mutant form into the pcDNA5/FRT/TO vector. The shRNA constructs were made by cloning targeting sequences into pLKO.1/puro plasmid. The sequence targeting NEAT1 is: 5′-CGCTTGTAATCCCAGCACTTT-3′, and 5′-GCTGAGGCAGAAGAATCACTT-3′; targeting MIOX is: 5′-CCAGGTGATGAAGTTTAACAA-3′, and 5′-GTTCAACAAGTTCGACCTCTA-3′; targeting p53 is: 5′-CACCATCCACTACAACTACAT-3′, and 5′-GAGGGATGTTTGGGAGATGTA-3′; targeting HIF-1α is: 5′-CCAGTTATGATTGTGAAGTTA-3′, and 5′-TGCTCTTTGTGGTTGGATCTA-3′; targeting ERα is: 5′-CTACAGGCCAAATTCAGATAA-3′, and 5′-GCAGGATTGTTGTGGCTACTA-3′; targeting Oct4 is: 5′-ACTATGCACAACGAGAGGATT-3′, and 5′-CCCTCACTTCACTGCACTGTA-3′; targeting ATF2 is: 5′-GCGAAATCTGTGGTTGTAAAT-3′, and 5′-CCATCCTCTAACAGGCCAATT-3′; targeting SFPQ is: 5′-CGCCTGTAATCCCAGCACTTT-3′, and 5′-GCCTGTAATCCCAGCACTTTA-3′; targeting NONO is: 5′-CAGGCGAAGTCTTCATTCATA-3′, and 5′-GCAGGCGAAGTCTTCATTCAT-3′; targeting PSPC1 is: 5′-GAGCTGCTAGAGCAAGCATTT-3′, and 5′-CCATACAAGCAGTTTATGGAT-3′. All constructs were confirmed by sequencing.

### Cell viability assay

Cell viability was evaluated using the cell counting kit-8 (CCK-8) (#96992, Sigma) according to the manufacturer’s instructions. Cells were seeded in 96-well plates (10,000 cells/well) and treated with erastin, RSL3, or dimethylsulfoxide (DMSO) for 12 h. Each well was replaced with 100 μL fresh complete medium containing 10 μL of the CCK-8 solution and then incubated at 37  °C with 5% CO_2_ for 1 h. The assay is based on the reduction of the highly water-soluble tetrazolium salt WST-8 [2- (2-methoxy-4-nitrophenyl)–3-(4-nitrophenyl)−5(2,4-disulfophenyl)−2H-tetrazolium, monosodium salt] to generate a water-soluble methyldye in the presence of an electron carrier. The absorbance at 450 nm is directly proportional to the number of living cells in the culture. Absorbance at 450 nm is proportional to the number of living cells in the culture.

### Malondialdehyde (MDA) assay

The relative MDA concentration in cell lysate was determined by using the Lipid Peroxidation Assay Kit (ab118970) purchased from Abcam according to the manufacturer’s instruction. In short, the MDA in cells or tissues reacted with thiobarbituric acid (TBA) to form a colored MDA-TBA adduct whose absorbance is measured at 532 nm.

### Glutathione (GSH) assay

The relative GSH concentration in samples was measured using the Glutathione Assay Kit (CS0260, Sigma). Cells were seeded in 10 cm plates (5 × 10^6^ per plate) and treated with erastin, RSL3, or DMSO for 12 h. Cells were washed with ice-cold PBS and lysed in GSH assay buffer. And GSH levels were measured according to the manufacturer’s instruction. The measurement of GSH used a kinetic assay in which catalytic amounts (nmoles) of GSH caused a continuous reduction of 5,5′-dithiobis (2-nitrobenzoic acid) to 5-thio-2-nitrobenzoic acid. The reaction rate was proportional to the concentration of glutathione. The yellow product (5-thio-2-nitrobenzoic acid) was measured spectrophotometrically at 412 nm.

### Nicotinamide adenine dinucleotide phosphate (NADPH) measurement

NADPH levels were analyzed using an NADP/NADPH Assay Kit (ab65349, Abcam). Cells were seeded in 10 cm^2^ plates and treated with DMSO, erastin, or RSL3 for 12 h. Cells were lysed in a 200 μL NADP/NADPH extraction buffer. The samples were then briefly vortexed and centrifuged at 14,000 g for 5 min. The supernatants were passed through spin columns provided in the kit and centrifuged at 10,000 *g* for 40 min. The filtered samples were then heat-treated at 60 °C for 30 min to decompose NADP. Fifty microliter standards or samples were added into the individual wells of a 96-well plate, which had preadded 100 μL reaction mix (98 μL NADP cycling buffer and 2 μL NADP cycling enzyme mix). Then, 10 μL NADPH developer (ab65349, Abcam) was added to each well, and the reaction extended for 2 h. After the reaction period, colorimetric reading was measured spectrophotometrically at 450 nm. The concentrations of these metabolites were normalized to the amount of total protein.

### Lipid ROS measurement

Lipid ROS level was analyzed by flow cytometry using BODIPY-C11 (Thermo, #D3861) dye. Cells were seeded at a density of 2.5 × 10^5^ per well in a six-well dish and grown overnight and were treated with erastin, RSL3, or DMSO for 12 h. At the end of the treatment, the culture medium was removed, and cells were washed once with PBS. Cells were then stained with 2 mL medium containing 5 µM of BODIPY-C11 and incubated at 37 °C for 20 min in the dark. Cells were washed twice with PBS to remove excess labeling mixture followed by resuspending in 500 μL fresh PBS (DPBS, Gibco). The cell suspension was filtered through a 0.4 μM cell filter and subjected to flow cytometric analysis to detect the amount of intracellular lipid ROS. The fluorescence intensities of cells per sample were determined by flow cytometry using the BD FACSAria cytometer (BD Biosciences). A minimum of 10,000 cells was analyzed for each sample. Data analysis was evaluated using the FlowJo Software.

### Iron assay

Intracellular ferrous iron (Fe^2+^) level was determined using the iron assay kit (ab83366, Abcam) according to the manufacturer’s instructions. Cells were seeded in a 10 cm^2^ plate (5 × 10^6^ cells per plate) and treated with erastin, RSL3, or DMSO for 12 h. Cells were collected and washed in ice-cold PBS and homogenized in 5× volumes of iron assay buffer on ice, then centrifuged (13,000 × *g*, 10 min) at 4 °C to remove insoluble material. Collect the supernatant and add iron reducer to each sample before mixing, and incubated at room temperature for 30 min. Then, 100 μL of the iron probe was added to each sample, mixing and incubate the reaction for 1 h at room temperature in the dark. The absorbance at 593 nm was measured immediately using a colorimetric microplate reader.

### RNA extraction, cDNA synthesis, and real-time PCR analysis

Total RNAs from cultured cells were extracted with TRIzol (Thermo), and cDNA synthesis was performed using a reverse transcription kit (Promega) according to the manufacturer’s instructions. Real-time PCR was analyzed on the CFX96 touch real-time PCR detection system using a standard protocol from the SYBR Green PCR Kit (Bio-rad). The expression of lncRNA and protein coding genes were normalized to that of Actin. Primer sequences used for qRT-PCR assays were: NEAT1_1 forward, 5’-GCGAGGTGCCTTTACTACAT-3’ and NEAT1_1 reverse, 5′- TGGAACCCAGAAGACAGA-3′; NEAT1_2 forward, 5′-TCCGAGGAAGATGTAAGG-3′ and NEAT1_2 reverse, 5′-TCTGTGGAATGAGGCAAC-3′; MIOX forward, 5′-CTGGATGGGCTGGTGGAT-3′ and MIOX reverse, 5′-GAAGGTGGAGTCGCAGAAAA-3′; Actin forward, 5′-GCTCGTCGTCGACAACGGCT-3′ and Actin reverse, 5′-CAAACATGATCTGGCTCATCTTCTC-3. To verify the miR-362-3p expression, total RNA was isolated using the Rneasy Mini Kit (Qiagen 74104), and 1 µg of total RNA was used for cDNA synthesis by using TaqMan™ MicroRNA Reverse Transcription Kit (Thermo 4366596). The qPCR analysis was performed by TaqMan miRNA assays and standardized to small nuclear RNA (Rnu6) (Thermo 4426961). All PCR assays were repeated three times.

### RNA–Seq and differentially expressed gene (DEG) analysis

For RNA sequencing, purified RNA from erastin- and RSL3-induced HepG2 cells and control cells was used for library construction with Illumina TruSeq RNA Sample Prep Kit (FC-122-1001) and then sequenced with Illumina HiSeq 2000. Raw reads were aligned to the human genome GRCh37/hg19 by TopHat2 [19]. DEGs between treatment and control samples were identified with DEseq2. A heatmap clustered by k-means was used to show DEGs or transcripts. Finally, differentially expressed genes between control and treatment samples were obtained by paired t-test with *p* value < 0.05. In addition, transcriptome analysis of NEAT1 knockdown and control HepG2 cells induced by erastin or DMSO was conducted to create another RNA-Seq library, all other operations as described above.

### Chromatin immunoprecipitation (ChIP) qPCR

ChIP was performed according to the manufacture’s instruction for the MAGnify™ Chromatin IP System (Thermo, 492024). In short, HepG2 cells treated with erastin or RSL3 for 8 h were cross-linked with 1% formaldehyde (Sigma-Aldrich) at room temperature for 10 min and quenched with 125 mM glycine for 5 min. Then, cross-linked cells were washed twice with cold PBS, lysed by lysis buffer, and sonicated using Model 120 Sonic Dismembrator (ThermoFisher, FB120A220) to obtain 200–500 bp chromatin fragments. Antibodies were coupled to magnetic beads (Millipore) overnight at 4 °C. Anti-p53 monoclonal antibody (ab1101, Abcam) was used for immunoprecipitation. Chromatin-protein complexes were incubated overnight at 4 °C with antibody-coupled magnetic beads. Next, immunoprecipitated chromatin complexes were precipitated and washed. The crosslinking of eluted chromatin was completely reversed by incubation overnight at 65 °C with proteinase K (ThermoFisher). The purification of the precipitated DNA was performed using the ChIP DNA Clean & Concentrator Kits (Zymo Research). Quantification of purified DNA samples was subjected to qPCR with the primer sets placed adjacent to the promoter of the listed genes. Primer sequences are as follows: promoter region of NEAT1, forward 5′-AATCACCCCACCCCAACC-3′, and reverse 5′-ACATTTCGCCTGCGTCTG -3′; GAPDH was used as a control with the following primer set, forward 5′-GGCAGCACAGCCCACAGGTT-3′, and reverse 5′-ATCGTGACCTTCCGTGCAGAAAC-3′.

### Luciferase reporter assay

The promoter sequence of NEAT1 was amplified from the genomic DNA of HEK293T cells by PCR and cloned into the pGL-4.23 vector. To generate the NEAT1 mutant construct, we used the NEB Q5 site-directed mutagenesis kit to delete the binding sequences (GAGCAAGCCTGGGCTTGCCA). HepG2 cells were cultured in six-well plates in advance, and co-transfected with the promoter construct along with the pRL-CMV Renilla luciferase reporter plasmid as internal control with polyfect transfection reagent (Qiagen 301107). The PCR primers of NEAT1 promoter are forward, 5′-GGTACCACTCCACTGCCCAGTACT-3′ and reverse 5′-AAGCTTACCGAGAAGAAGCTGGTG-3′.

The sequence of NEAT1 (or MIOX 3′UTR) was inserted into the psiCHECK2 basic construct (Promega) (NEAT1 forward, 5′-CTCGAGATTGTTTTGCTTTGCTAC-3′, and NEAT1 reverse 5′-GCGGCCGCGCAGCGAAGGATGCTGAT-3′; MIOX 3′UTR forward, 5′-CTCGAGAGCCCCACTGGGTGTTAC-3′, and MIOX 3′UTR reverse 5′-GCGGCCGCTGCCCAGCCTGCATTTGT-3′). To generate the NEAT1-MUT and 3′UTR mutants of MIOX, the binding site (GGUGUGU) was changed to (TCTAGAT) using the site-directed mutagenesis kit (NEB E0554). And HepG2 cells were cotransfected with a psiCHECK-2 luciferase reporter plasmid and miR-362-3p overexpression or indicated constructs. All the clones were verified by DNA sequencing. 12 h after transfection, we replaced the transfection medium with a complete culture medium. At 48 h post-transfection, cells were lysed with passive lysis buffer, and the reporter gene expression was measured using the dual-luciferase reporter assay system (Promega E1910). Measurements were made on the Beckman-Coulter DTX880. At least four replicates with three independent experiments were performed, and the transfection efficiency was normalized with Renilla luciferase.

### RNA immunoprecipitation (RIP)

RIP assay was conducted using the Magna RIP™ Kit (17-700, Millipore Corporation, Billerica, MA) according to the manufacturer’s instructions. Briefly, HepG2 cells were harvested and lysed in RIP lysis buffer. Then, cell lysates were incubated with magnetic beads which were conjugated with anti-Ago2 antibody (ab186733, Abcam) or corresponding negative control IgG antibody (Millipore) at 4 °C for 6 h. The beads were washed and incubated with proteinase K to remove proteins. Finally, the immunoprecipitated RNA was purified and analyzed by qRT-PCR.

### Immunoblotting

For immunoblotting, cells were trypsinized and washed with ice-cold PBS, lysed in lysis buffer (50 mM Tris-HCl PH 8.0, 1% SDS, 1 mM EDTA, 5 mM DTT, 10 mM PMSF, 1 mM NaF, 1 mM Na_3_VO_4_, and protease inhibitor cocktail), and then denatured in boiling water for 10 min. The cellular lysates were centrifuged (13,000 × *g*, 30 min), and protein concentration was determined using a BCA assay (ThermoFisher, 23225). Cell lysates were resolved by SDS-PAGE and transferred to polyvinylidene difluoride membranes (Bio-Rad). Membranes were blocked with 5% skim milk and incubated with the indicated antibodies at 4 °C overnight. HRP-conjugated goat secondary antibodies were used (1:5000, Invitrogen). Then membranes were washed three times with TBST followed by secondary antibody incubation for 2 h at room temperature. Immunodetection was achieved with the chemiluminescence reagent (Thermo) and detected by a GE ECL machine.

### Northern blotting

Total RNA was extracted using TRIzol (Invitrogen) and separated by 1% denaturing agarose gel electrophoresis. The RNA was transferred to a positively charged nylon membrane (77016, Biodyne™ B Nylon Membrane, Thermo, MA, USA) according to standard protocols, followed by UV cross-linking. Digoxigenin-labeled probes were achieved using the DIG Northern Starter Kit (12039672910, Roche) and hybridized with the DIG Easy Hyb reagent at 68 °C overnight. The membrane was washed and detected using the reagents provided in the DIG Northern starter kit according to the manufacturer’s description. Primers for northern blot probes are as follows: NEAT1_1 forward, 5′-CATGGTGCTCTCAGAACCCACCTC-3′, and NEAT1_1 reverse 5′- TAATACGACTCACTATAGGGGAGGAAGTGGCTAGACCTGACGCTA-3′; NEAT1_2 forward, 5′-AATGTTGGTCCTCTCCTCATGTGCC-3′, and NEAT1_1 reverse 5′- TAATACGACTCACTATAGGGTCGCTGTGTACAATGTTCTGCTCCC-3′.

### Lentivirus production

For lentivirus production, HEK293T cells were transfected with pLKO.1/puro plasmids together with pCMV-dR8.91 and pCMV-VSV-G packing plasmids using Calcium Phosphate Transfection Kits (Clontech). Viral particles were collected 48 h after transfection, filtered with 0.45 µm sterile filter, and concentrated by ultracentrifugation at 4 °C (24,000 rpm, 2 h, Beckman-Coulter ultracentrifuge XL- 100 K).

### UALCAN database

UALCAN (http://ualcan.path.uab.edu/) is a publicly available database for analyzing the cancer genome atlas transcriptome data. Using the UALCAN database, we analyzed the expression of RNA-Seq DEGs in HCC tissues and normal liver tissues. *p* value < 0.05 was considered to indicate a statistically significant result.

### The miRNA target prediction

Firstly, DIANA-LncBase V2 (https://diana.e-ce.uth.gr/lncbasev3/interactions) and starbase v2.0 (http://starbase.sysu.edu.cn/starbase2/mirLncRNA.php) were used to analyze the relationships between the dysregulated lncRNAs and miRNAs. The predicted targets of lncRNA from the two websites were compared, and the overlapping miRNAs were used as candidates. Secondly, miRTarBase (http://mirtarbase.cuhk.edu.cn/php/index.php) and targetscan (http://www.targetscan.org/vert_72/) were employed to decode the relationships between the dysregulated miRNAs and mRNAs. Similarly, overlapping miRNAs were used as candidates. Finally, the predicted lncRNA-miRNA pairs and miRNA–mRNA pairs were combined to construct lncRNA–miRNA–mRNA regulatory networks based on ceRNA regulatory mechanisms. miRNA-mRNA binding site was obtained from targetscan and lncRNA-miRNA binding sites were predicted by starbase.

### Colony formation assay

For the colony formation assay, cells were seeded in 60 mm dishes. At 70–80% confluency, cells were treated with erastin, RSL3, or DMSO for 12 h. Cells were trypsinized, counted, and replated in appropriate dilutions in a six-well plate for colony formation. After 2 weeks of incubation, colonies were fixed and stained in a mixture of 6% glutaraldehyde (Amresco) and 0.5% crystal violet for 1 h, carefully rinsed with tap water, and dried at room temperature. Plating efficiency was determined for each cell line, and the surviving fraction was calculated based on the number of colonies that arise after treatment. Each experiment was repeated three times.

### Xenotransplantation experiments

NU/NU nude mice were purchased from Charles River (Beijing). All animal studies were performed following guidelines of the Institutional Animal Care and Use Committee of Beijing Institute of Biotechnology. For xenograft models, HepG2 or HuH-7 cells (5 × 10^6^ cells per mouse) that were transfected with the NEAT1 vector or an empty vector were injected into the left posterior flanks of 7-week-old immunodeficient female nude mice. The tumors were measured every 4 days, and tumor volume was calculated using the following formula: volume = (*L* × *W*^2^)/2, among which *L* and *W* are the longest and shortest diameters, respectively. When tumors reached a volume of ~50 mm^3^, mice were randomly allocated into groups and treated with erastin or RSL3 via intraperitoneal injection for 20 days. Mice were then sacrificed, the xenograft tumors were excised and weighted for immunohistochemistry assays. The erastin was dissolved in 5% DMSO + corn oil (C8267, Sigma). To better dissolve erastin, we warmed the tube at 37 °C water base and shake it gently. All animal procedures were performed in accordance with institutional guidelines.

### Immunohistochemistry (IHC) staining

Paraffin-embedded, formalin-fixed xenograft mouse tissues were immunostained for MIOX and 4-HNE. In brief, tumor tissues were fixed in 4% (v/v) formaldehyde in PBS, embedded in paraffin, and cut into 3 μm thick sections. Tissue slides were dewaxing, rehydrated, antigen retrieval, and blocking. After antigen retrieval, the sections were incubated with the primary antibodies overnight at 4 °C in a moist chamber and washed three times with PBST. Sections were incubated with HRP-conjugated secondary antibody for 15 min at room temperature, washed three times with PBST, and then stained with DAB and hematoxylin. Sections were observed under an optical microscope at 200×. Experiments were repeated at least three times.

### In situ hybridization (ISH)

For ISH, DIG-labeled locked nucleic acid microRNA probe (Exiqon) was used to measure miR-362-3p expression in xenograft mouse tissues. Briefly, paraffin slides were deparaffinized with xylene and dehydrated with ethanol. Then incubated with proteinase K for 10 min at 37 °C and hybridized with miR-362-3p probe at 52  °C overnight. After stringency washes, staining was developed with an anti-DIG-POD antibody and DAB complex. Finally, the slides were counterstained with hematoxylin. The miR-362-3p probe was: 5′-AACACACCUAUUCAAGGAUUCA-3′.

### Statistical analysis

All statistical analyses were performed using the Graphpad Prism 8.0 software. The data are presented as mean ± SD from multiple individual experiments each performed in triplicate and each experiment was repeated at least three times. Student’s *t* test (two-tailed) was applied to compare difference between two groups. One-way analysis of variance (ANOVA) was used for comparison among the different groups. When ANOVA was significant, post hoc testing of differences between groups was performed using the Least Significant Difference test. *p* value < 0.05 were considered statistically significant.

## Results

### NEAT1 is upregulated in erastin- and RSL3-induced ferroptosis

**Fig. 1:**
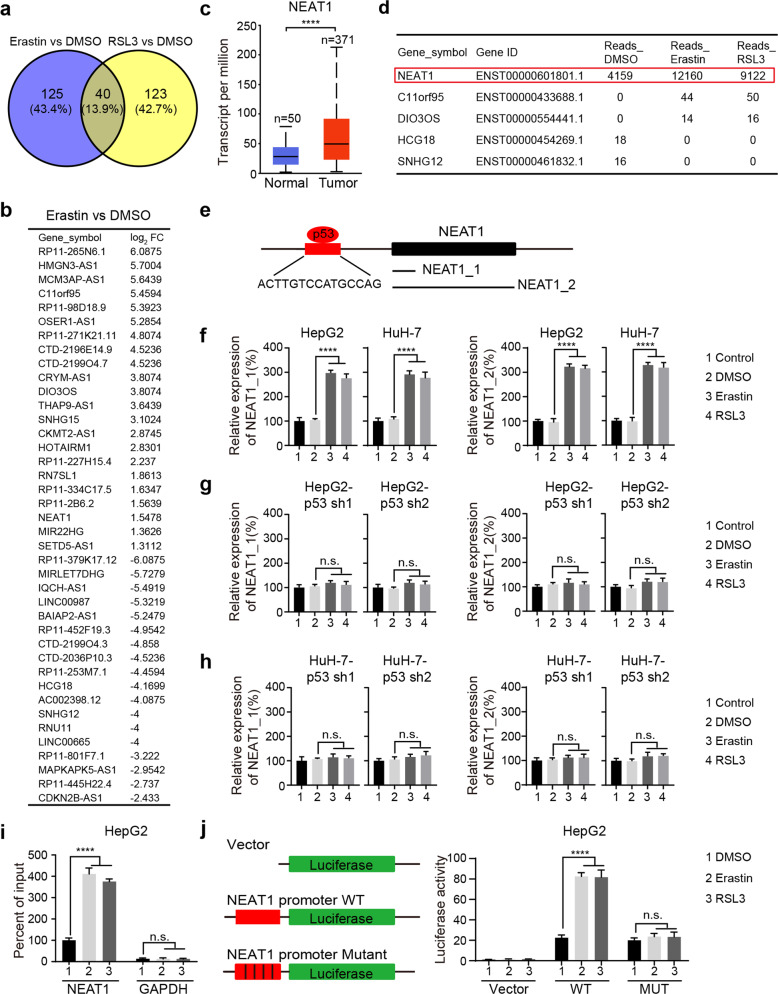
Induced expression of NEAT1 in ferroptosis depends on p53. **a** Veen map shows the number of genes that were significantly differentially expressed in HepG2 cells treated with erastin or RSL3 versus HepG2 cells treated with DMSO (more than onefold and *p* < 0.05). **b** The log2 FC values of the 40 overlapping genes were compared between erastin group and DMSO group. **c** NEAT1 is significantly upregulated in HCC tumor tissues. The relative expression of NEAT1 in tumor tissue (*n* = 371) and corresponding normal tissue (*n* = 50) was analyzed using UALCAN, and the data were derived from the cancer genome atlas database. **d** The read counts of five lncRNAs in HepG2 cells treated with DMSO, erastin, or RSL3. **e** Schematic representation of the p53 binding site in the promoter region of NEAT1 and two isoforms of NEAT1. The p53 binding site (ACTTGTCCATGCCAG) is marked red, and the two isoforms (NEAT1_1 and NEAT1_2) are transcribed from the same locus. **f** Erastin and RSL3 induced the expression of NEAT1_1 and NEAT1_2 in HepG2 and HuH-7 cells. Cells were treated with erastin (5 µM) or RSL3 (0.5 µM) for 12 h; the mRNA levels of NEAT1_1 and NEAT1_2 were measured by qRT-PCR. Control group represents non-transfected cells. **g, h** Knockdown of p53 inhibited the upregulation of NEAT1_1 and NEAT1_2 induced by erastin and RSL3. HepG2 and HuH-7 cells stably expressing p53 shRNA constructs were treated with erastin (5 µM) or RSL3 (0.5 µM) for 12 h, and the mRNA levels of NEAT1_1 and NEAT1_2 were measured by qRT-PCR. Control group represents non-transfected cells. **i** ChIP-qPCR showed that erastin or RSL3 treatment increased the binding of p53 to the NEAT1 promoter. HepG2 cell was treated with erastin (5 µM) or RSL3 (0.5 µM) for 8 h, the DNA fragments purified after co-immunoprecipitation were amplified by qPCR, GAPDH was the negative control. **j** Luciferase reporter assay showed that erastin or RSL3 treatment increased the activity of NEAT1 promoter, but not the mutant form. The NEAT1 promoter-reporter constructs were transfected into HepG2 cells for 24 h, and the dual-luciferase activity was determined after erastin (5 µM) or RSL3 (0.5 µM) treatment for 12 h. Data shown represent mean ± SD from three independent experiments. ****p < 0.0001; n.s., not significant.

To identify lncRNAs involved in ferroptosis, an HCC cell line, HepG2, was treated with ferroptosis-inducing agents for transcriptome analysis with RNA-Seq. As shown, following erastin and RSL3 treatments, 40 of the lncRNAs resulted in significant changes in gene expression from both treatments (|log_2_ FC| > 1, *p* value < 0.05) (Supplementary Table 1), and 125 or 123 of the lncRNAs affected by either erastin or RSL3, respectively (|log_2_ FC| > 1, *p* value < 0.05) (Fig. 1a, b, Supplementary Fig. 1a and Supplementary Table 2). In general, genes that are highly expressed in tumor cells play an important role in tumorigenesis and treatment [20]. Therefore, we mined the UALCAN database [21] (http://ualcan.path.uab.edu/) against our list of differentially expressed genes and found five interesting lncRNAs (NEAT1, C11orf95, DIO3OS, HCG18, SNHG12) which are significantly expressed in HCC compared with normal tissues (Fig. 1c and Supplementary Fig. 1b). Among them, the expression of NEAT1 was significantly higher than that of other lncRNAs (Fig. 1d), and it has been reported to be involved in tumor development, progression and treatment. Therefore, we further study the function and mechanism of NEAT1 in ferroptosis.

### Erastin- and RSL3-induced expression of NEAT1 is mediated by p53

NEAT1 has two isoforms, NEAT1_1 is constitutively expressed in most human tissues, whereas the expression of NEAT1_2 is tissue-specific. However, the biological function of NEAT1 is mainly derived from the NEAT1_2 isoform [22]. To detect the expression of two isoforms, we designed primers to generate two amplicons, primer pair 1 detects the expression of both NEAT1_1 and NEAT1_2, and primer pair 2 only detects NEAT1_2 (Fig. 1e). As shown in Fig. 1f, both transcripts were upregulated in erastin- and RSL3-treated HCC cells, indicating the expression of NEAT1_1 and NEAT1_2 were induced in ferroptosis.

Previous reports have shown that NEAT1 is regulated by various transcription factors under different conditions, such as p53, HIF-1α, ERα, Oct4, and ATF2 [23–26]. To further investigate which transcription factor is involved in the upregulation of NEAT1, we generated stable cell lines with these transcription factors knocked down by shRNA constructs. We found that depletion of p53 eliminated the upregulation of NEAT1 in HepG2 and HuH-7 cells (Fig. 1g, h), suggesting that erastin- and RSL3-induced expression of NEAT1 is mediated by p53. In contrast, knockdown of other transcription factors had no significant effect on the upregulation of NEAT1 induced by erastin and RSL3 (Supplementary Fig. 1c–f). These data further proved that p53 plays an essential role in regulating the expression of NEAT1 in ferroptosis.

To further test whether p53 directly regulates the transcription of NEAT1, we analyzed the promoter region of NEAT1 using JASPAR program (http://jaspar.genereg.net/cgi-bin/jaspar_db.pl) and found a binding sequence for p53 (GAGCAAGCCTGGGCTTGCCA). Then, we performed chromatin immunoprecipitation (ChIP) assays and found that erastin and RSL3 treatment promoted p53 binding to the promoter region of NEAT1 (Fig. 1i). In addition, we observed increased promoter activity after erastin or RSL3 treatment of NEAT1-WT promoter, but not the mutant form without p53 binding site (Fig. 1j). Furthermore, knockdown of p53 abolished erastin- or RSL3-induced transcriptional activation of NEAT1 promoter, but not the basal transcriptional activity (Supplementary Fig. 1g). Taken together, these results indicate that p53 directly binds to the promoter of NEAT1 and promotes NEAT1 expression after ferroptosis-inducing conditions.

### NEAT1 promotes erastin- and RSL3-induced ferroptosis

**Fig. 2:**
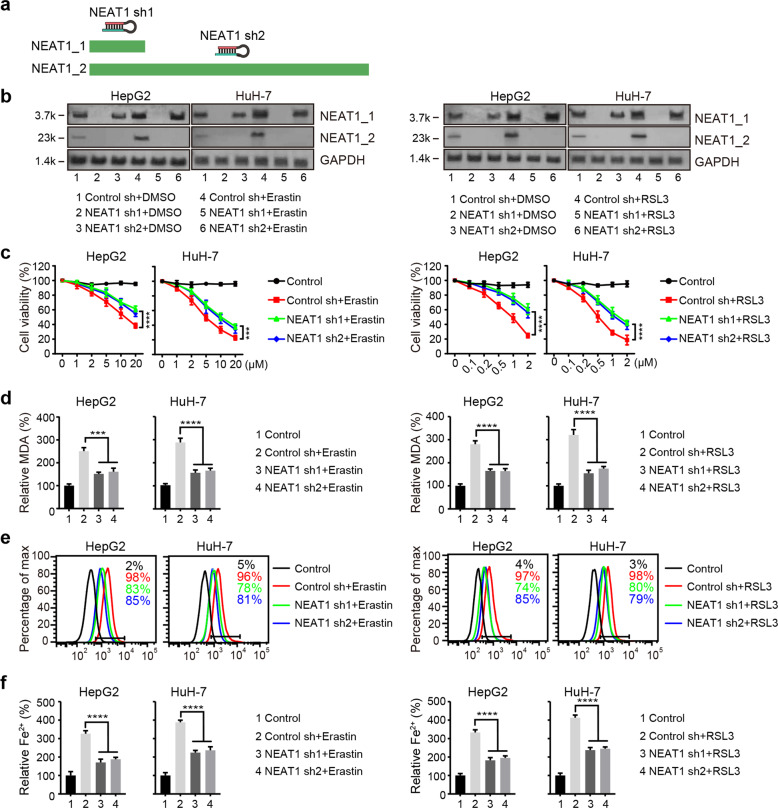
NEAT1 promotes erastin- and RSL3-induced ferroptosis. **a** Schematic diagram of the positions of two NEAT1 shRNA sequences. **b** The relative expression levels of NEAT1_1 and NEAT1_2 in HepG2 and HuH-7 cells transfected with indicated constructs were detected by northern blot. See Supplementary figure 7 for uncropped data. Knockdown of NEAT1 suppressed erastin- and RSL3-induced ferroptosis. Cells transfected with indicated constructs were treated with erastin (1–20 µM) or RSL3 (0.1–2 µM) for 12 h, cell viability was determined with a CCK-8 kit (**c**), lipid formation was measured by MDA assay (**d**), lipid ROS accumulation was analyzed by flow cytometry with C11-BODIPY staining (**e**), and the intracellular Fe^2+^ was measured by iron detection assay (**f**). Control group represents non-transfected cells. Data shown represent mean ± SD from three independent experiments. ***p < 0.001; ****p < 0.0001; n.s., not significant.

To investigate whether NEAT1 regulates ferroptosis, we constructed two NEAT1 specific shRNAs. NEAT1 shRNA1 knocked down both NEAT1_1 and NEAT1_2, while NEAT1 shRNA2 only knocked down NEAT1_2 without affecting the expression of NEAT1_1 (Fig. 2a, b and Supplementary Fig. 2b). Next, we generated HepG2 and HuH-7 cell lines stably expressing two different NEAT1 specific shRNAs and treated them with erastin or RSL3. As shown in Fig. 2c, knockdown of both NEAT1 isoforms or NEAT1_2 alone in HepG2 or HuH-7 cells significantly inhibited erastin- and RSL3-induced cell death. Because Fe^2+^ and lipid ROS are essential for ferroptosis process, and malondialdehyde (MDA) is an important end product of lipid ROS [27], we further measured their concentrations in erastin- and RSL3-treated HCC cells and found that knockdown of NEAT1 significantly inhibited the accumulation of MDA, lipid ROS and Fe^2+^ (Fig. 2d, e, f). Collectively, these data indicate that NEAT1 promotes erastin- and RSL3-induced ferroptosis in HCC cells.

### NEAT1 regulates ferroptosis by modulating the expression of MIOX

**Fig. 3:**
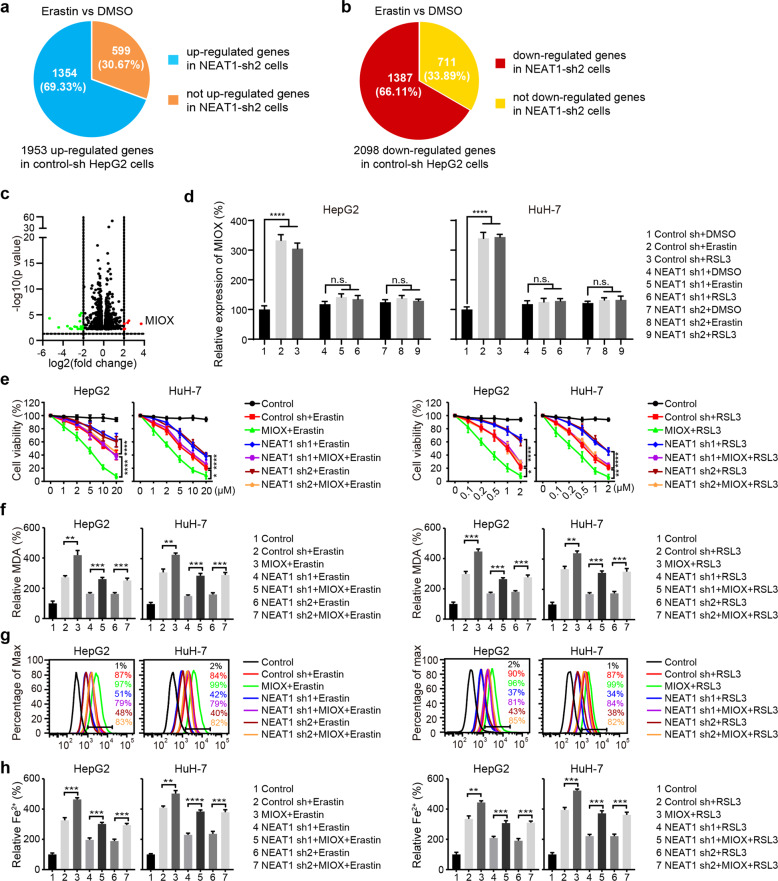
NEAT1 promotes ferroptosis by regulating MIOX. **a** Veen maps show that 1953 genes are upregulated in erastin treated HepG2 cells transfected with control shRNA. Note that 1354 genes are still upregulated in cells transfected with NEAT1 shRNA, while 599 genes are not upregulated. **b** Veen maps show that 2098 genes are downregulated in erastin treated HepG2 cells transfected with control shRNA. Note that 1387 genes are still downregulated in cells transfected with NEAT1 shRNA, while 711 genes are not downregulated. **c** The volcano plot reflects the differentially expressed genes regulated by NEAT1 in **a** and **b**. Red marks, log2 FC > 2 and *p* value < 0.05; green marks, log2 FC < −2 and *p* value < 0.05. **d** Erastin- and RSL3-induced expression of MIOX is dependent on NEAT1. HepG2 and HuH-7 cells transfected with indicated constructs were treated with erastin (5 µM) or RSL3 (0.5 µM) for 12 h, and the mRNA level of MIOX was measured by qRT-PCR. Overexpression of MIOX enhanced erastin- and RSL3-induced ferroptosis, which was inhibited by NEAT1 shRNA. HepG2 and HuH-7 cells transfected with indicated constructs were treated with erastin (1–20 µM) or RSL3 (0.1–2 µM) for 12 h, cell viability was determined with a CCK-8 kit (**e**), lipid formation was measured by MDA assay (**f**), lipid ROS accumulation was analyzed by flow cytometry with C11-BODIPY staining (**g**), and the intracellular Fe^2+^ was measured by iron detection assay (**h**). Control group represents non-transfected cells. Data shown represent mean ± SD from three independent experiments. *p < 0.05; **p < 0.01; ***p < 0.001; ****p < 0.0001; n.s., not significant.

To further elucidate the mechanism of NEAT1 during ferroptosis, we searched for NEAT1 target genes by comparing the gene expression differences caused by knocking down NEAT1. As shown in Fig. 3a and b, there were 1953 upregulated genes and 2098 downregulated genes after erastin treatment in HepG2 cells transfected with control shRNA construct (*p* value < 0.05) (Supplementary Table 3). However, in HepG2 cells transfected with the NEAT1 shRNA construct, 599 upregulated genes and 711 downregulated genes were unresponsive to erastin (*p* value < 0.05) (Supplementary Table 4), indicating that the modified expression of these genes in ferroptosis was dependent on NEAT1 (Fig. 3a, b). Moreover, we identified 6 upregulated genes and 15 downregulated genes with more significant modification (|log_2_ FC| > 2 and *p* value < 0.05) (Fig. 3c, Supplementary Fig. 2a) and found that MIOX was the most highly upregulated gene after erastin treatment. Meanwhile, qRT-PCR detection showed that neither erastin nor RSL3 could induce the upregulation of MIOX in NEAT1 knockdown cells, which further confirmed that the induction of MIOX expression in ferroptosis was dependent on NEAT1 (Fig. 3d). Similarly, knockdown of MIOX inhibited erastin- and RSL3-induced cell death, as well as ferroptotic events, including the accumulation of MDA, lipid ROS, and Fe^2+^ (Supplementary Fig. 2d-g). Overexpression of MIOX rescued erastin- and RSL3-induced ferroptotic cell death inhibited by NEAT1 deficiency (Fig. 3e). Consistently, overexpression of MIOX increased the intracellular levels of MDA, lipid ROS, and Fe^2+^ in the NEAT1 knockdown cells (Fig. 3f–h). Taken together, these results suggest that NEAT1 promotes erastin- and RSL3-induced ferroptosis by regulating the expression of MIOX.

### MIOX promotes ferroptosis by regulating Fe^2+^, GSH, and NADPH

**Fig. 4:**
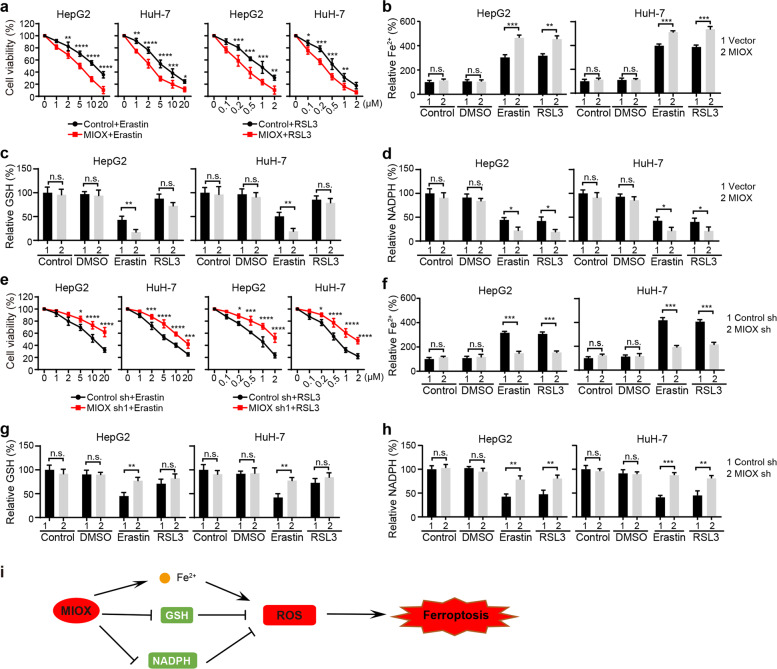
MIOX promotes ferroptosis by regulating Fe^2+^, GSH, and NADPH. Overexpression of MIOX increased erastin- and RSL3-induced cell death (**a**), Fe^2+^ accumulation (**b**). **c, d** Overexpression of MIOX suppressed the concentration of GSH in erastin treated cells and NADPH levels after erastin or RSL3 treatment. Knockdown of MIOX suppressed erastin or RSL3-induced cell death (**e**), intracellular level of Fe^2+^ (**f**). Knockdown of MIOX increased the concentration of GSH in erastin treated cells (**g**) and NADPH levels after erastin or RSL3 treatment (**h**). Control group represents non-transfected cells. Data shown represent mean ± SD from three independent experiments. *p < 0.05; **p < 0.01; ***p < 0.001; ****p < 0.0001; n.s., not significant. **i** Schematic of the mechanism by which MIOX**-**regulates ferroptosis.

A previous study has demonstrated that overexpression of MIOX exacerbates cisplatin-induced acute kidney injury by regulating iron accumulation, GSH activity, and NADPH levels [28]. To test whether this mechanism is conserved in erastin- and RSL3-induced ferroptosis in HCC cells, we overexpressed MIOX in HCC cells and found MIOX promoted erastin- and RSL3-induced cell death (Fig. 4a and Supplementary Fig. 3a). On the contrary, knockdown of MIOX or d-glucaric acid (MIOX inhibitor) treatment suppressed erastin- and RSL3-induced cell death (Fig. 4e and Supplementary Fig. 3b). We further detected the intracellular level of Fe^2+^. As shown in Fig. 4b, the intracellular concentration of iron was significantly higher in MIOX-overexpressing cells after erastin and RSL3 treatment. As the main intracellular antioxidant, GSH scavenges intracellular lipid hydrogen peroxide to prevent ferroptosis [29]. We found that overexpression of MIOX further reduced the GSH levels in both HepG2 and HuH-7 cells after treatment with erastin (Fig. 4c). Similarly, NADPH contributes to the elimination of lipid ROS and regulates the sensitivity of cells to ferroptosis, which was also reduced in MIOX-overexpressing cells (Fig. 4d). In contrast, either depletion of MIOX or d-glucaric acid treatment inhibited the accumulation of Fe^2+^, the reduction of NADPH, and the reduction of GSH induced by erastin in HepG2 and HuH-7 cells (Fig. 4f-h and Supplementary Fig. 3c–e). In summary, MIOX promotes ferroptosis by regulating the intracellular levels of Fe^2+^, GSH, and NADPH (Fig. 4i).

### MiR-362-3p is involved in the regulation of MIOX by NEAT1

NEAT1 is an essential lncRNA that regulates basic biological processes, such as cell proliferation and apoptosis [30]. Functionally, NEAT1 either forms paraspeckles with more than 40 proteins such as PSPC1, NONO, and SFPQ, or acts as a ceRNA to regulate miRNA expression [31]. Paraspeckles are multifunction nuclear structures that accelerate the proliferation or inhibit apoptosis through sequestering of transcriptionally active proteins as well as RNA transcripts [25]. Besides NEAT1, proteins SFPQ, NONO, and PSPC1 are important constituent proteins of paraspeckles. To test whether NEAT1 regulates the ferroptosis process by forming paraspeckles, we generated HepG2 cell lines expressing shRNA constructs specific to SFPQ, NONO, and PSPC1, and treated them with erastin or RSL3. Depletion of SFPQ, NONO, or PSPC1 did not significantly affect erastin- and RSL3-induced cell death, lipid ROS accumulation, and Fe^2+^ level, suggesting that NEAT1 may not regulate ferroptosis by forming paraspeckles (Supplementary Fig. 4a–i).

**Fig. 5:**
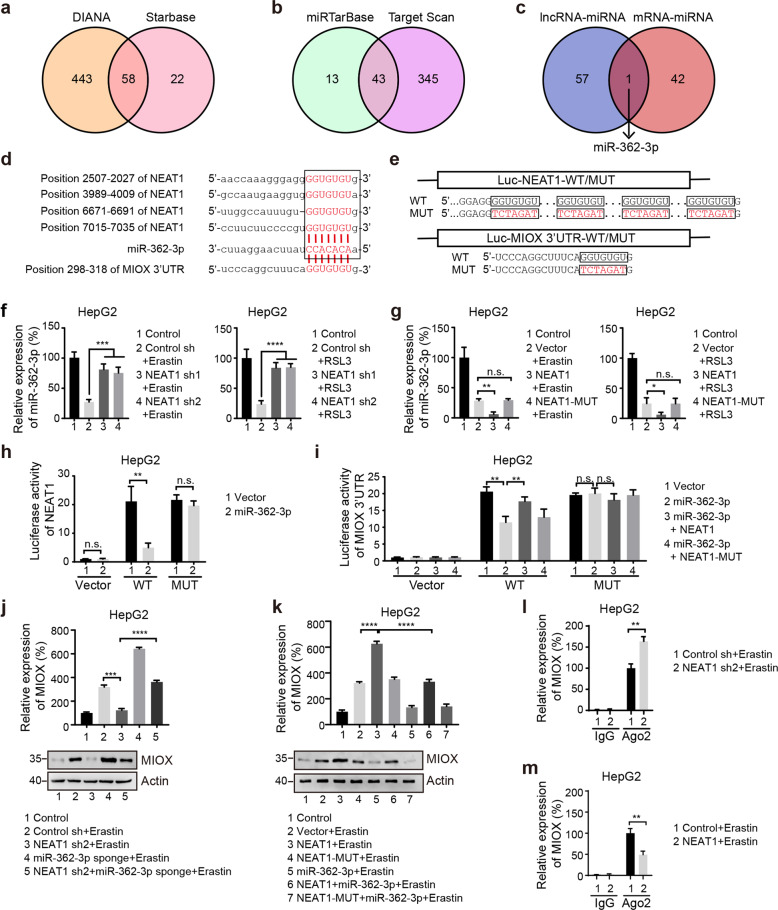
MiR-362-3p interacts with NEAT1 and MIOX. **a** A total of 58 overlapping miRNAs interacting with NEAT1 were predicted by DIANA and Starbase databases. **b** A total of 43 overlapping miRNAs interacting with MIOX were predicted by miRTarBase and Target Scan databases. **c** MiR-362-3p is the only overlapping miRNA in figure a and b. **d** Sequence alignment of NEAT1, MIOX, and miR-362-3p. NEAT1 contains four binding sequences (GGUGUGU) of miR-362-3p. **e** Schematic representation of wild type and mutant sequences of NEAT1 and MIOX 3’UTR. Knockdown of NEAT1 promoted miR-362-3p expression in ferroptosis (**f**), whereas overexpression of NEAT1, but not the mutant form, suppressed miR-362-3p (**g**). HepG2 cells transfected with indicated constructs were treated with erastin (5 µM) or RSL3 (0.5 µM) for 12 h, and the mRNA level of miR-362-3p was measured by qRT-PCR. Control group represents non-transfected cells. **h** MiR-362-3p inhibits the expression of a NEAT1-luciferase reporter in HepG2 cells, but not the mutant reporter. **i** Overexpression of wild-type NEAT1, but not the mutant form, increased the expression of a MIOX 3’UTR-luciferase reporter which was suppressed by miR-362-3p. Knockdown of miR-362-3p increased the expression of MIOX inhibited by NEAT1 shRNA (**j**), whereas overexpression of miR-362-3p decreased the expression of MIOX promoted by NEAT1 (**k**). HepG2 cells transfected with indicated constructs were treated with erastin (5 µM) for 12 h. The expression of MIOX was detected by qRT-PCR and western blot. See Supplementary figure 8 for uncropped western blot image. Control group represents non-transfected cells. **l, m** The interaction between MIOX and miR-362-3p was affected by the expression level of NEAT1, which was verified by RIP assay using anti-Ago2 antibody, followed by qPCR to detected the MIOX level. Data shown represent mean ± SD from three independent experiments. *p < 0.05; **p < 0.01; ***p < 0.001; ****p < 0.0001; n.s., not significant.

Accumulating evidence suggests that lncRNAs regulate target genes by binding to miRNAs as ceRNA [32]. To determine whether NEAT1 functions as ceRNA during ferroptosis, we used predictive tools to find 58 potential miRNA binding sites in NEAT1 using both starbase v2.0 [33] and DIANA-LncBase V2 [34] (Fig. 5a). As MIOX is proved to be the target of NEAT1 in ferroptosis, we then use miRTarBase [35] and targetscan [36] to find potential miRNA binding sites in MIOX, 43 potential MIOX-binding miRNAs were present in both of the two databases (Fig. 5b). By comparing the miRNAs predicted by two methods, we found that miR-362-3p was the only overlapping miRNA (Fig. 5c), which may mediate the regulation of MIOX and ferroptosis by NEAT1.

### MiR-362-3p interacts with NEAT1 and MIOX

Using two publicly available bioinformatics tools targetscan and starbase, we found that the NEAT1 and 3′UTR of MIOX contain the putative miR-362-3p binding site (Fig. 5d). To confirm the bioinformatics-based predictions, we cloned the cDNA sequence of NEAT1 and the 3′UTR sequence of MIOX into the psiCHECK-2 vector. We next introduced several mismatch mutations into seed sequences to generate mutant reporter vectors and performed luciferase activity assays (Fig. 5e). As shown in Fig. 5f and supplementary figure 5a, knockdown of NEAT1 increased the expression level of miR-362-3p in HepG2 and HuH-7 cells after erastin or RSL3 treatment. In contrast, overexpression of NEAT1, but not the mutant form, decreased the expression level of miR-362-3p (Fig. 5g and Supplementary Fig. 5b). Consistently, overexpression of miR-362-3p significantly inhibited the transcriptional activity of wild-type NEAT1, but not the mutant form (Fig. 5h). Together, these results suggest that NEAT1 regulates miR-362-3p.

To determine whether MIOX is a direct target of miR-362-3p, we co-transfected the miR-362-3p overexpression constructs with the reporter vector containing the 3′UTR of MIOX. As shown in Fig. 5i, overexpression of miR-362-3p dramatically attenuated the luciferase activity of the reporter vector containing the wild-type 3′UTR of MIOX. In contrast, mutation of the seed sequence blocked the inhibitory effect of miR-362-3p (Fig. 5i). Moreover, we found that the effect of miR-362-3p on the activity of MIOX 3’UTR could be partially retrieved by overexpressed NEAT1 (rather than mutant form), suggesting that NEAT1 occupies miR-362-3p to avoid binding to the 3′UTR of MIOX (Fig. 5i).

To further demonstrate that miR-362-3p is involved in cross-regulation between MIOX and NEAT1, we evaluated the expression of MIOX. Knockdown of NEAT1 suppressed the mRNA and protein expression of MIOX in HepG2 cells treated with erastin or RSL3. In contrast, knockdown of miR-362-3p markedly increased the mRNA and protein expression of MIOX inhibited by NEAT1 shRNA (Fig. 5j and Supplementary Fig. 5c). Furthermore, overexpression of wild-type NEAT1, but not mutant form, increased the mRNA and protein expression of MIOX. Overexpression of miR-362-3p significantly inhibited the mRNA and protein expression of MIOX induced by NEAT1 (Fig. 5k and Supplementary Fig. 5d).

MiRNAs suppress the translation and degradation of mRNAs in an Ago2-dependent manner. To test whether the interaction between MIOX and miR-362-3p was affected by NEAT1, RIP experiments were performed on NEAT1 overexpression or knockdown HepG2 cells. As shown in Fig. 5l and Supplementary Fig. 5e, the mRNA level of MIOX pulled down by Ago2–miR-362-3p complex was significantly enriched in NEAT1 depleted HepG2 cells, while NEAT1 overexpression markedly decreased the mRNA level of MIOX (Fig. 5m and Supplementary Fig. 5f). Taken together, these results suggest that NEAT1 positively modulates MIOX expression via sponging miR-362-3p.

### MiR-362-3p inhibits ferroptosis by regulating MIOX

**Fig. 6:**
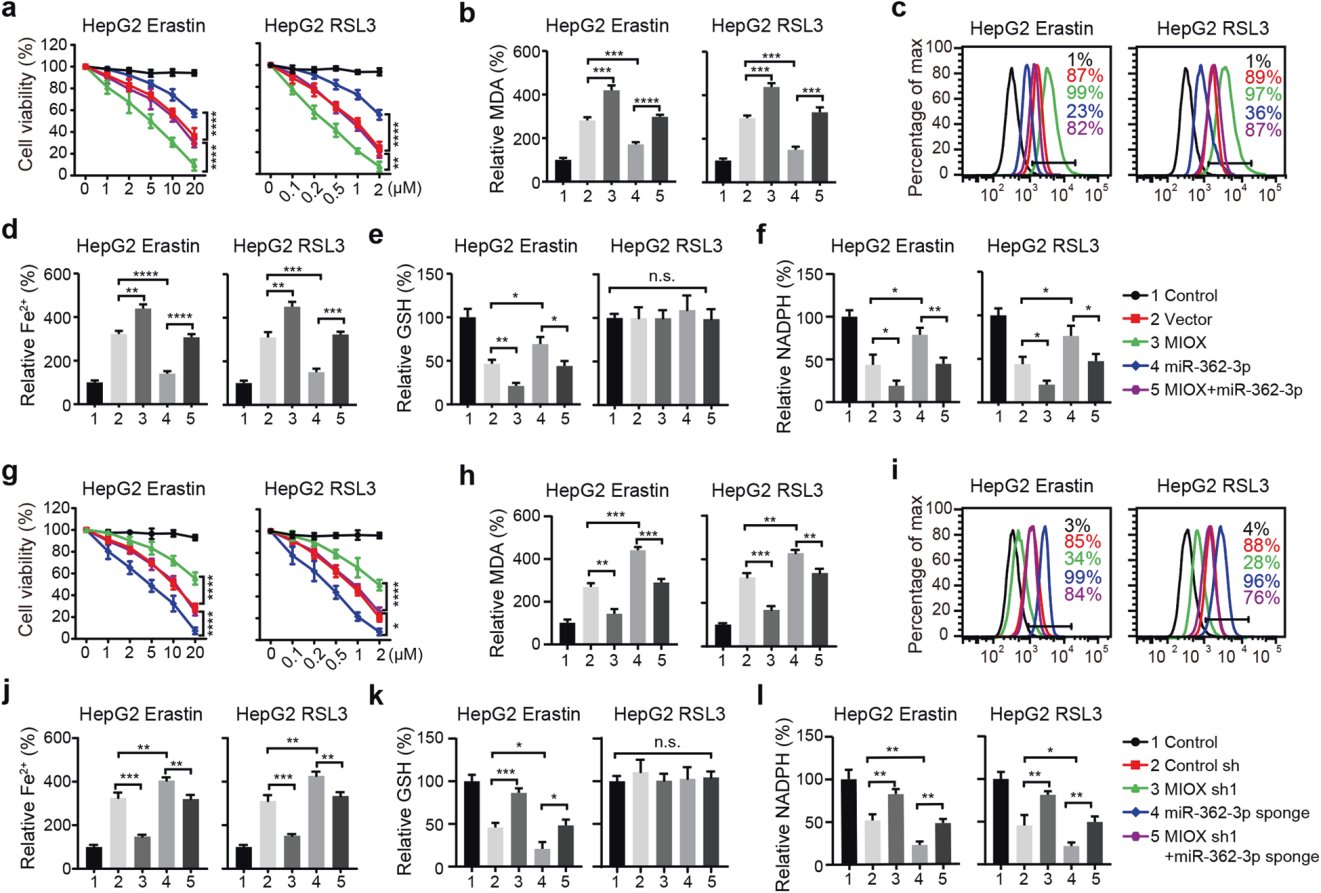
MiR-362-3p inhibits ferroptosis by regulating MIOX. Overexpression of MIOX promotes erastin- and RSL3-induced ferroptosis, which was inhibited by miR-362-3p. HepG2 cells transfected with indicated constructs were treated with erastin (1–20 µM) or RSL3 (0.1–2 µM) for 12 h, cell viability was determined with a CCK-8 kit (**a**), lipid formation was measured by MDA assay (**b**), lipid ROS accumulation was analyzed by flow cytometry with C11-BODIPY staining (**c**), the intracellular Fe^2+^ was measured by iron detection assay (**d**), GSH concentration in the cells was measured using a glutathione assay kit (**e**), and NADPH level was determined using an NADPH assay kit (**f**). Knockdown of MIOX inhibits erastin- and RSL3-induced ferroptosis, which was enhanced by miR-362-3p sponge. HepG2 cells transfected with indicated constructs were treated with erastin (1–20 µM) or RSL3 (0.1–2 µM) for 12 h, cell viability was determined with a CCK-8 kit (**g**), lipid formation was measured by MDA assay (**h**), lipid ROS accumulation was analyzed by flow cytometry with C11-BODIPY staining (**i**), the intracellular Fe^2+^ was measured by iron detection assay (**j**), GSH concentration in the cells was measured using a glutathione assay kit (**k**) and NADPH level was determined using an NADPH assay kit (**l**). Control group represents non-transfected cells. Data shown represent mean ± SD from three independent experiments. *p < 0.05; **p < 0.01; ***p < 0.001; ****p < 0.0001; n.s., not significant.

To analyze whether miR-362-3p modulates ferroptosis through its effects on MIOX, we performed rescue experiments by overexpressing MIOX. The coding sequence of MIOX was cloned into an expressing vector, which did not have the miR-362-3p binding sequence, making it resistant to miRNA-mediated downregulation. HepG2 cells transfected with miR-362-3p significantly suppressed erastin- and RSL3-induced cell death and reduced the promoting effect of MIOX (Fig. 6a). Meanwhile, the accumulation of MDA, lipid ROS, and Fe^2+^ were also partially restored upon overexpression of MIOX (Fig. 6b–d). Further, the overexpression of MIOX suppressed GSH and NADPH’s intracellular levels induced by miR-362-3p (Fig. 6e, f). In addition, HepG2 cells transfected with miR-362-3p sponge significantly promoted erastin- and RSL3-induced cell death and related ferroptotic events, including lipid ROS production, iron accumulation, NADPH suppression and erastin-induced GSH depletion (Fig. 6g–l). Knockdown of MIOX suppressed erastin- and RSL3-induced ferroptosis promoted by miR-362-3p sponge (Fig. 6g–l). These results demonstrate that miR-362-3p regulates ferroptosis by modulating the expression of MIOX.

### NEAT1 overexpression promotes ferroptosis induced by erastin or RSL3 in vitro and in vivo

**Fig. 7:**
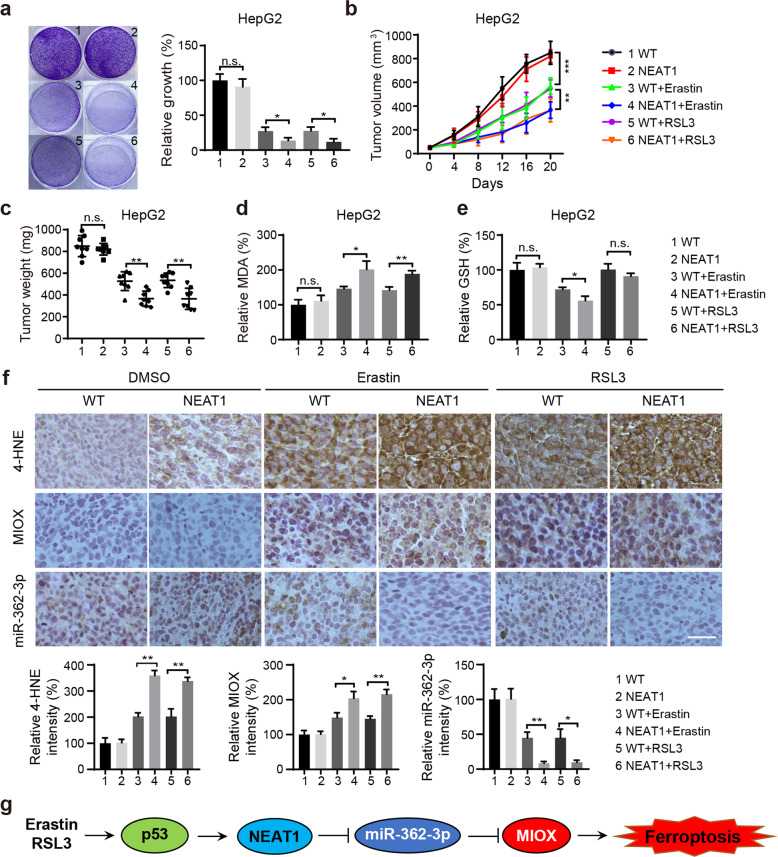
Overexpression of NEAT1 inhibits erastin or RSL3 resistance in vitro and in vivo. **a** Overexpression of NEAT1 promoted erastin- and RSL3-induced ferroptosis detected by the colony formation assay. HepG2 cells were treated with DMSO, erastin (5 µM), or RSL3 (0.5 µM) for 12 h and cultured for 10 days without erastin or RSL3, and the number of cell colonies was calculated. Overexpression of NEAT1 enhanced erastin- and RSL3-induced ferroptosis in vivo. Corresponding HepG2 cells were cultured and injected subcutaneously into 7-week-old immunodeficient mice (8 mice per group) at 5 × 10^6^ cells per mouse, and erastin (15 mg/kg, twice every other day) or RSL3 (10 mg/kg, twice every other day) were injected intraperitoneally when the tumor volume of the mice reached 50 mm^3^. Tumor volume **b** was measured every 4 days, tumor weight **c** was measured on day 20. The relative levels of MDA **d** and GSH **e** were measured. **f** The expression level of 4NHE, MIOX and miR-362-3p of tumor xenografts was detected by immunohistochemical analysis and in situ hybridization staining. of miR-362-3p. Quantitative analysis of intensity was also shown. Scale bar, 50 µm. The experiment was repeated twice independently with similar results. Data shown represent mean ± SD from three independent experiments. *p < 0.05; **p < 0.01; ***p < 0.001; n.s., not significant. **g** Schematic diagram of NEAT1/miR-362-3p/MIOX axis regulating ferroptosis in HCC cells. NEAT1 positively regulates MIOX expression through the sponge to miR-362-3p, which promotes ferroptotic cell death.

The observed effects of NEAT1 on ferroptosis suggest that the expression level of NEAT1 should modulate the anti-tumor activity of erastin and RSL3. Thus, we stably overexpressed NEAT1 in HepG2 and HuH-7 cells by a lentiviral vector, treated the cells with erastin or RSL3, and then measured its effect on cell viability by colony formation assay. We found that cells with high expression levels of NEAT1 were more sensitive to erastin- and RSL3-induced ferroptosis cell death (Fig. 7a and Supplementary Fig. 6a).

To further explore the effect of NEAT1 expression levels on the anticancer activity of erastin or RSL3 in vivo. HepG2 and HuH-7 cells with stable NEAT1 overexpression were subcutaneously injected into NU/NU nude mice. When the tumors reached at 50 mm^3^, mice were treated with erastin or RSL3 for 20 days. Compared with the control vector group, the size of tumors with increased expression of NEAT1 is markedly smaller than the control group treated with the same drug (Fig. 7b, c and Supplementary Fig. 6b, c), and exhibited the increased MDA levels and reduced erastin-induced GSH levels (Fig. 7d, e and Supplementary Fig. 6d, e). To further demonstrate the level of lipid peroxidation in tumor samples, we performed immunohistochemistry on 4-hydroxy-2-noneal (4-HNE). As expected, the staining for 4-HNE was markedly increased following treatment with erastin or RSL3 in NEAT1 overexpression cells (Fig. 7f and Supplementary Fig. 6f). Similarly, the expression of MIOX was significantly increased in NEAT1 overexpression cells. Moreover, ISH staining confirmed that the expression of miR-362-3p was reduced in NEAT1 overexpressing cells after treatment with erastin or RSL3 (Fig. 7f and Supplementary Fig. 6f). Together, NEAT1 modulated the anti-tumor activity of erastin and RSL3 in HCC cells in vivo.

## Discussion

In this study, we found that NEAT1, a bona fide target gene of p53, was significantly upregulated during erastin- or RSL3-induced ferroptosis in HCC cells. NEAT1 can competitively bind more miR-362-3p and thus leads to less miR-362-3p-mediated MIOX inhibition, thereby increasing the sensitivity of HCC cells to ferroptosis (Fig. 7g).

LncRNAs are regarded as one of the key regulators in cancer progression and drug resistance by regulating the expression of downstream genes and various biological processes. Increasing evidence indicates that ferroptosis has significant implications on various human diseases, including cancers [37]. In recent years, there have been many reports on the regulatory relationship between lncRNA and ferroptosis. lncRNA p53RRA interacts with G3BP1 to promotes ferroptosis and apoptosis in lung cancer cells via nuclear sequestration of p53 [38]. In lung cancer, lncRNA LINC00336 served as an endogenous sponge of miR-6852 to regulate the expression of cystathionine-β-synthase and inhibit ferroptosis [11]. Moreover, lncRNA OIP5-AS1 inhibits ferroptosis by targeting the miR-128-3p/SLC7A11 pathway in prostate cancer [39]. Here, we found that NEAT1 promoted ferroptosis by modulating the miR-362-3p/MIOX axis in HCC cells. As more and more lncRNAs associated with ferroptosis will be identified and studied, lncRNAs are likely to become important tumor therapeutic targets in the future.

p53 is an essential transcription factor that regulates cell growth, differentiation, proliferation, and death. p53 is a tumor suppressor; inactivation of p53 leads to tumorigenesis and progression. Recent studies found that p53 also plays an important role in ferroptosis in cancer cells by regulating several downstream targets, contributing to its tumor suppression function. SLC7A11 is an essential ferroptosis gene that mediates cystine uptake. There is a p53 binding sequence in the promoter of SLC7A11; p53 represses the expression of SLC7A11 and enhances erastin-induced ferroptosis [40]. Glutaminases2 (GLS2), the liver-type glutaminase in mitochondria, plays a critical role in regulating ferroptosis. GLS2 has been reported as a direct transcriptional target of p53; p53 binds to the promoter region of GLS2 gene to promote the conversion from glutamate to a-ketoglutarate, which induces ferroptosis upon the deprivation of amino acids [41]. Spermidine/spermine N1-acetyltransferase 1 was shown as a direct p53 target which leads to lipid peroxidation and ferroptosis upon ROS stress [42]. Our study found that p53 binds to the promoter region of NEAT1 to increase the transcription level in erastin- and RSL3-induced ferroptosis, which further demonstrates the critical role of p53 in ferroptosis. However, in some cases, p53 inhibits ferroptosis, and the acetylation modification and single-nucleotide polymorphisms of p53 also affect the function of p53 in ferroptosis [43–45]. All these indicate that we still lack a comprehensive understanding of the role and mechanism of p53 in ferroptosis, and further studies are needed to elucidate how p53 regulates ferroptosis as a promoter or inhibitor in different cells and diseases.

Recent studies have shown that NEAT1 is involved in various forms of cell death processes, such as apoptosis, pyroptosis, and autophagy. Knockdown of NEAT1 could promote imatinib-induced apoptosis in K562 cells [46]. In the innate immune response, upregulated NEAT1 disassociates from paraspeckles, leading to inflammasome activation and promoting IL-1β production and pyroptosis [47]. In addition, NEAT1 directly binds to miR-29b as a ceRNA to upregulated the expression of Atg9a and promotes autophagy [48]. Here, we found that NEAT1 promoted erastin- and RSL3-induced ferroptosis in HCC cells by binding to miR-362-3p to increase MIOX expression. Together, these studies suggest that NEAT1 is an important lncRNA that mediates the cross-regulation among apoptosis, pyroptosis, autophagy, and ferroptosis.

In addition, NEAT1 can affect the sensitivity of tumors to chemotherapeutic drugs, and patients with high NEAT1 expression often have a poor prognosis [13]. A recent study has shown that ferroptosis inducers can significantly improve the sensitivity of tumor cells to chemotherapy, suggesting the importance of ferroptosis in the treatment of drug-resistant tumors [49]. Further efforts to decipher the crucial role of NEAT1 in the interplay between apoptosis, autophagy, and ferroptosis have profound clinical implications since the evasion of cell death underlies tumorigenesis and drug resistance. Therefore, these efforts are necessary to improve our understanding of lncRNA in tumorigenesis and provide a potentially very useful therapeutic strategy for future treatment. Perhaps in the future, for patients with high NEAT1 expression, ferroptosis therapy in combination with other treatments can achieve better therapeutic results.

## Supplementary information

supplymentary materials

Supplymentary Table 1

Supplymentary Table 2

Supplymentary Table 3

Supplymentary Table 4

Raw Data

Checklist

## Data Availability

The datasets used and analyzed during the current study are available from the corresponding author on reasonable request.
