# Supplementary material for: Long noncoding RNA NEAT1 promotes ferroptosis by modulating the miR-362-3p/MIOX axis as a ceRNA
Source: Cell Death Differ. 2022 Mar 25;29(9):1850–63. doi: 10.1038/s41418-022-00970-9 (PMC9433379; doi:10.1038/s41418-022-00970-9)
Supplement: Supplementary file 1 — supplymentary materials [file 41418_2022_970_MOESM1_ESM.docx]

**Supplementary information for**

Long non-coding RNA NEAT1 promotes ferroptosis by modulating the miR-362-3p/MIOX axis as a ceRNA

Ying Zhang^1^, Meiying Luo^1^, Xiaohong Cui^2^, Douglas O’Connell^3^ and Yongfei Yang^1^

**This file includes:**

Supplementary figures 1 to 8

Supplementary figure legends 1 to 8

**Other supplementary files for this manuscript include the following:**

Supplementary tables 1 to 4 (separate files)


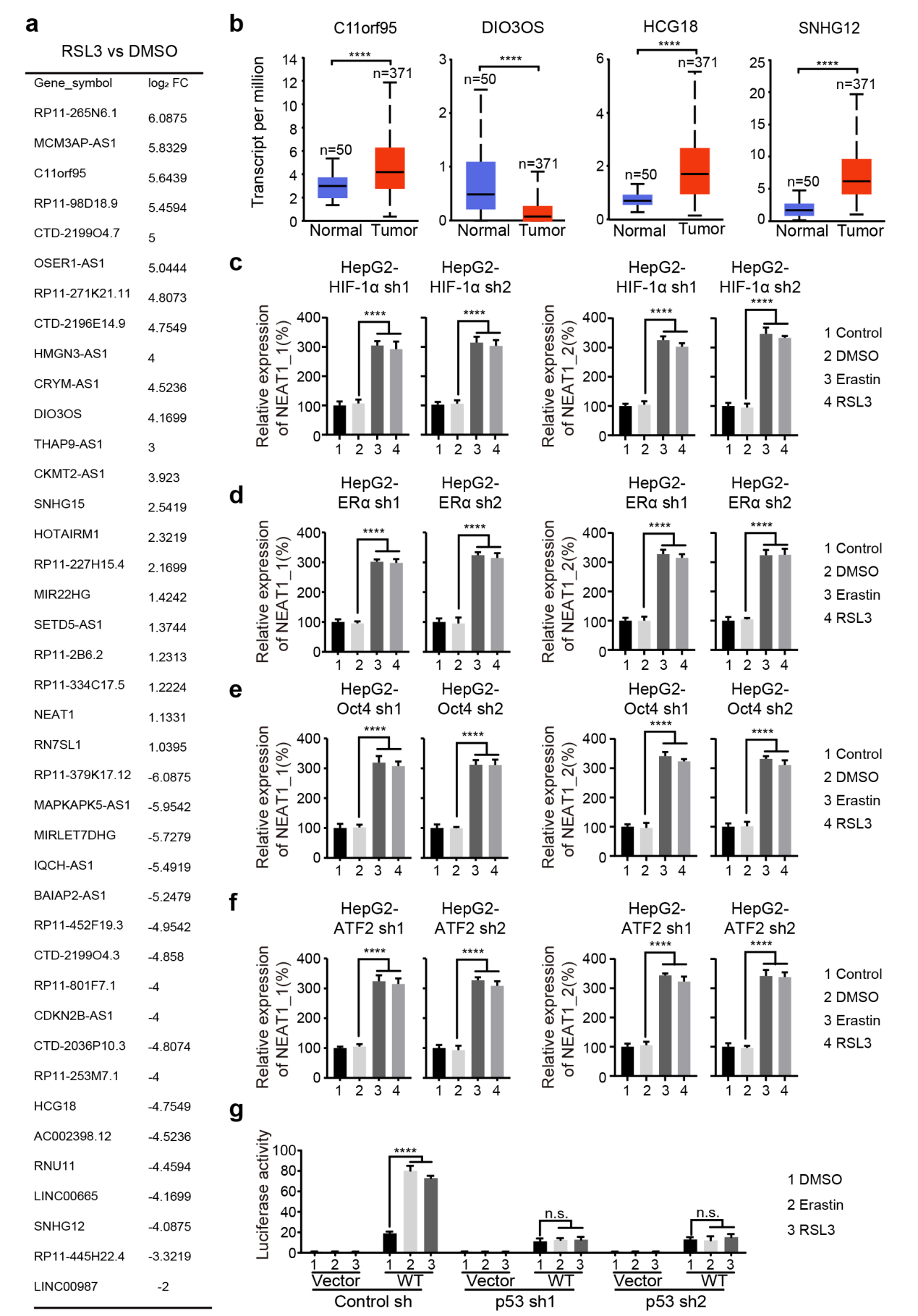


**Supplementary Fig. 1 The induced expression of NEAT1 in ferroptosis is independent of HIF-1α, ERα, Oct4, and ATF2. a** The log2 FC values of the 40 overlapping genes compared between DMSO group and RSL3 group. **b** The expression of HCG18, DIO3OS, C11ORF95, and SNHG12 are significantly different between LIHC tumor tissue and normal tissue. The relative expression of four lncRNAs in LIHC tumor tissue (n = 371) and corresponding normal tissue (n = 50) was analyzed using UALCAN, the data were derived from the cancer genome atlas database. **c-f** HIF-1α (**c**), ERα (**d**), Oct4 (**e**), and ATF2 (**f**) are not involved in the upregulation of NEAT1 induced by erastin or RSL3. HepG2 cells stably expressing indicated shRNA constructs were treated with erastin (5 µM) or RSL3 (0.5 µM) for 12 h, and the mRNA levels of NEAT1_1 and NEAT1_2 were measured by qRT-PCR. Control group represents non-transfected and non-treated cells. **g** Knockdown of p53 suppressed the luciferase activity of NEAT1 promoter induced by erastin or RSL3. The reporter constructs were transfected into indicated HepG2 cells for 24 h. Dual luciferase activity was then measured after treatment with erastin (5 µM) or RSL3 (0.5 µM) for 12 h. Data shown represent mean ± SD from three independent experiments. *****p* < 0.0001; n.s., not significant.


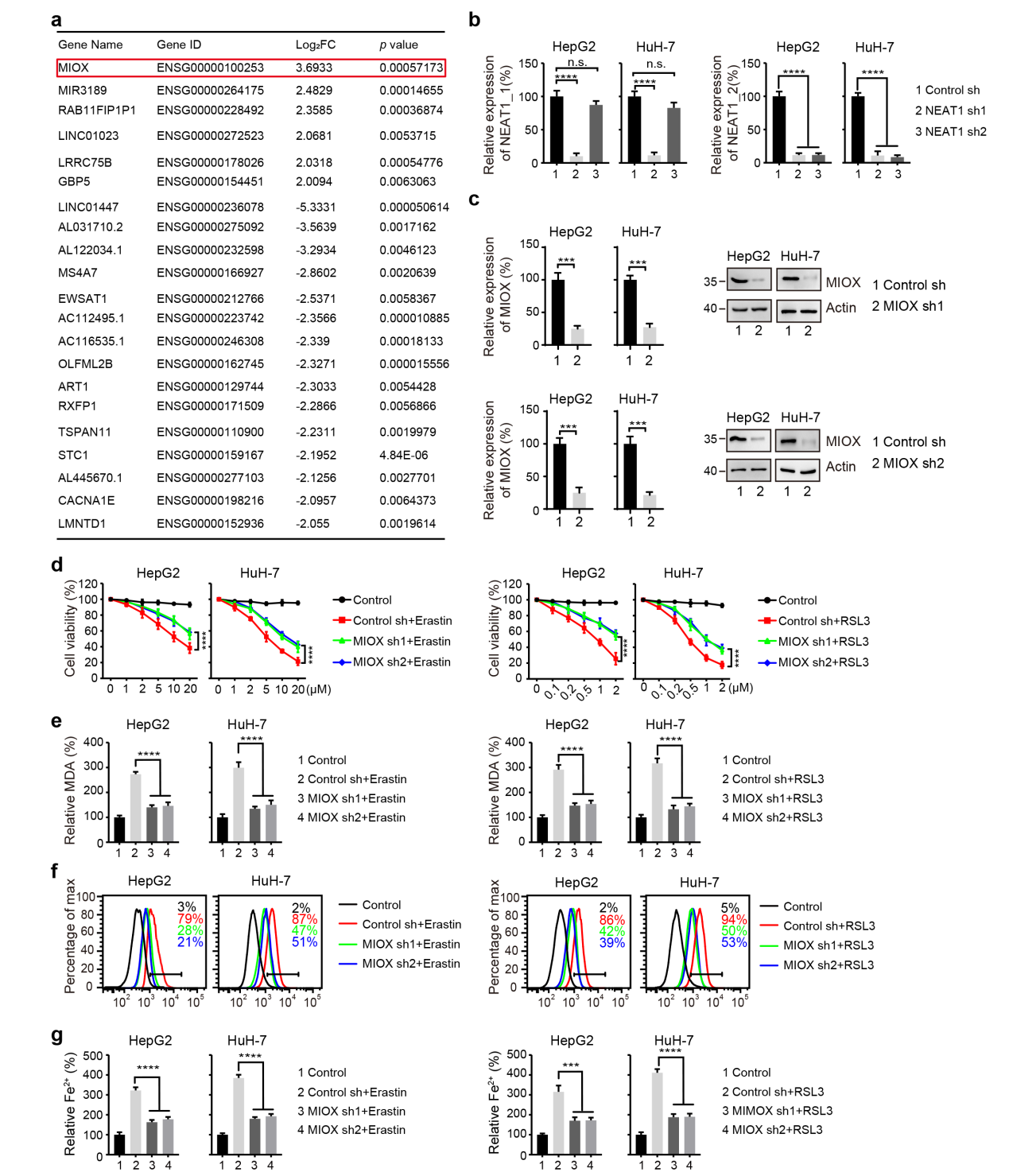


**Supplementary Fig. 2 MIOX promotes erastin- and RSL3-induced ferroptosis. a** Differentially expressed genes with |log2 FC| > 2 and *p* value < 0.05 in figure 3c volcano plot. **b** The relative expression levels of NEAT1_1 and NEAT1_2 in HepG2 and HuH-7 cells transfected with indicated constructs were detected by qRT-PCR. See Supplementary figure 8 for uncropped western blot image. **c** The knockdown efficiency of MIOX was measured by qRT-PCR and western blot. **d-g** Knockdown of MIOX suppressed erastin- or RSL3-induced ferroptosis. HepG2 and HuH-7 cells transfected with indicated constructs were treated with erastin (1-20 µM) or RSL3 (0.1-2 µM) for 12 h, cell viability was determined with a CCK-8 kit (**d**), lipid formation was measured by MDA assay (**e**), lipid ROS accumulation was analyzed by flow cytometry with C11-BODIPY staining (**f**), and the intracellular Fe^2+^ was measured by iron detection assay (**g**). Control group represents non-transfected and non-treated cells. Data shown represent mean ± SD from three independent experiments. ****p* < 0.001; *****p* < 0.0001; n.s., not significant.


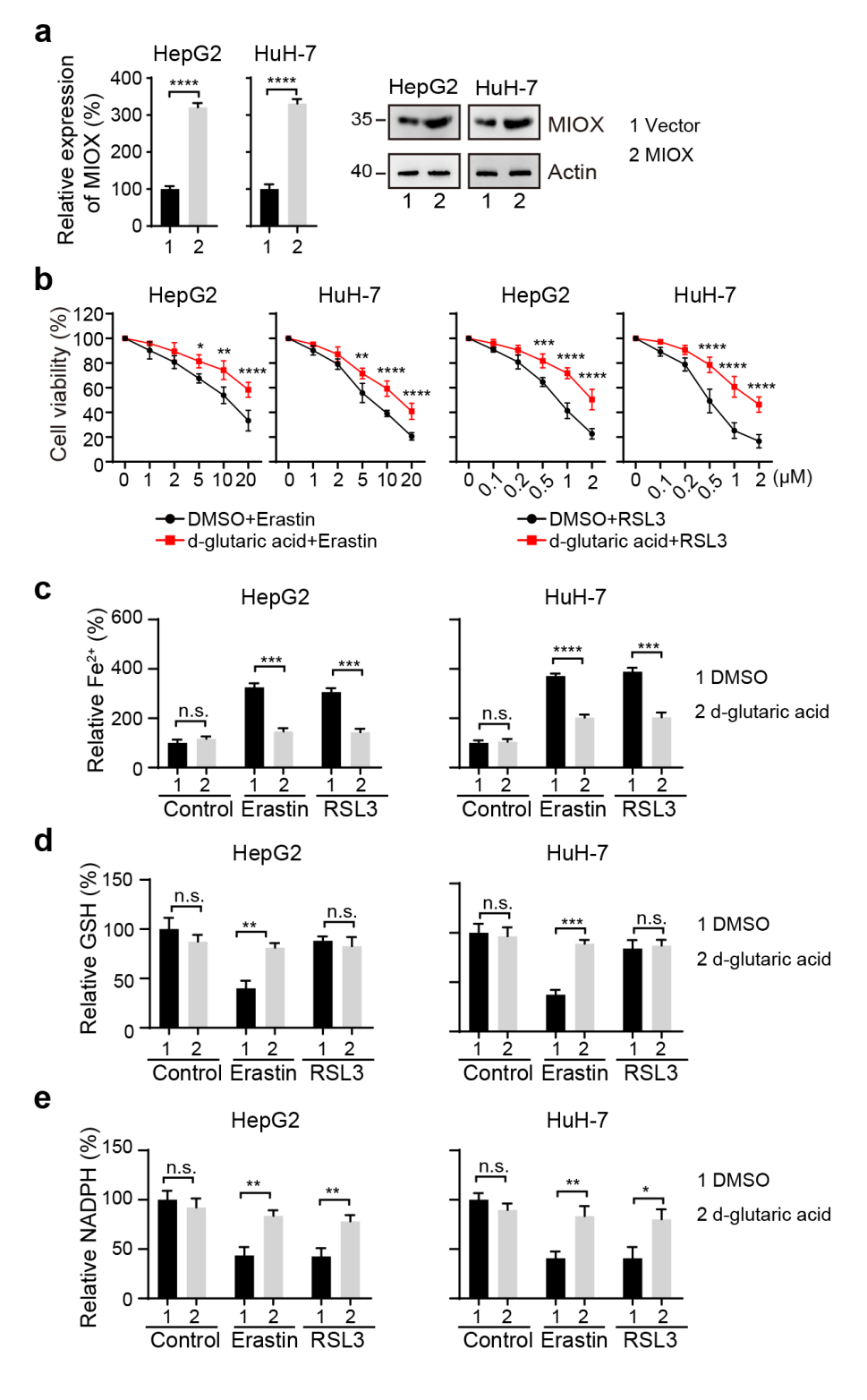


**Supplementary Fig. 3 D-glucaric acid suppress ferroptosis by regulating Fe^2+^, GSH, and NADPH. a** Relative expression level of MIOX overexpression was detected by qRT-PCR and western blot. See Supplementary figure 8 for uncropped western blot image. **b-e** D-glucaric acid suppressed erastin or RSL3-induced cell death (**b**), intracellular level of Fe^2+^ (**c**). D-glucaric acid increased the concentration of GSH in erastin treated cells (**d**) and NADPH levels after erastin or RSL3 treatment (**e**). Control group represents non-transfected and non-treated cells. Data shown represent mean ± SD from three independent experiments. *, *p* < 0.05; **, *p* < 0.01; ****p* < 0.001; *****p* < 0.0001; n.s., not significant.


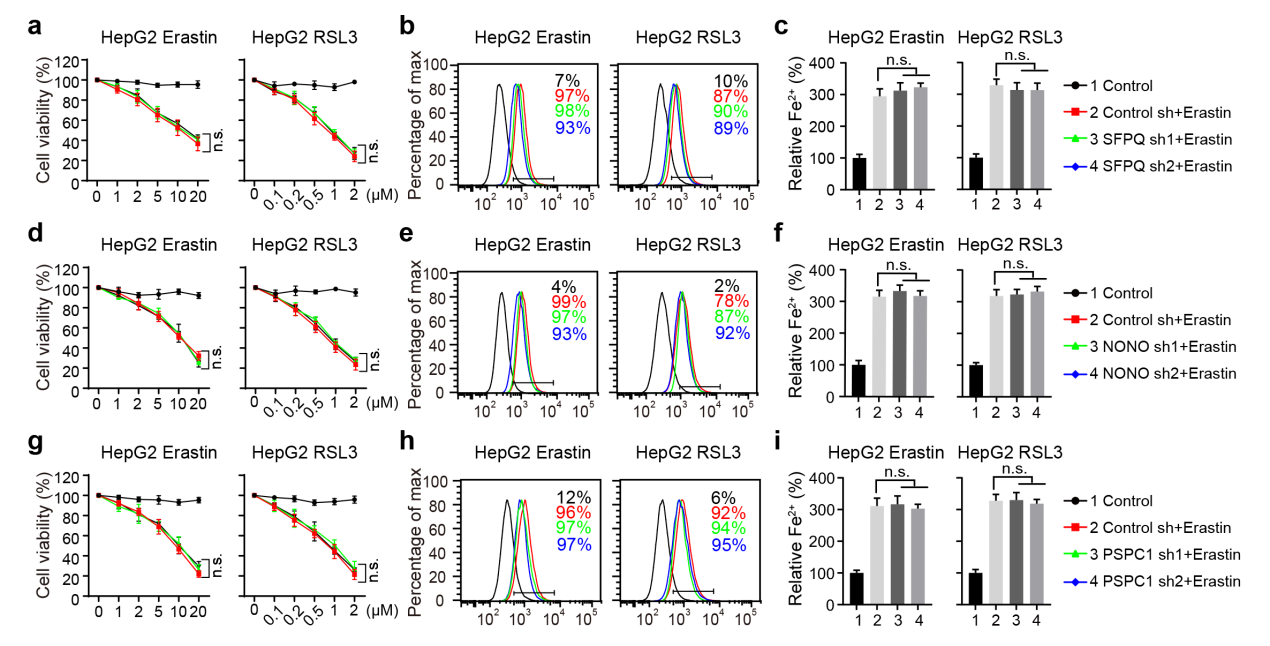


**Supplementary Fig. 4 The essential paraspeckle genes do not affect erastin- and RSL3-induced ferroptosis.** Cells transfected with indicated constructs were treated with erastin (1-20 µM) or RSL3 (0.1-2 µM) for 12 h, cell viability was determined with a CCK-8 kit (**a, d, q**), lipid ROS accumulation was analyzed by flow cytometry with C11-BODIPY staining (**b, e, h**), and the intracellular Fe^2+^ was measured by iron detection assay (**c, f, i**). Control group represents non-transfected and non-treated cells. Data shown represent mean ± SD from three independent experiments. n.s., not significant.


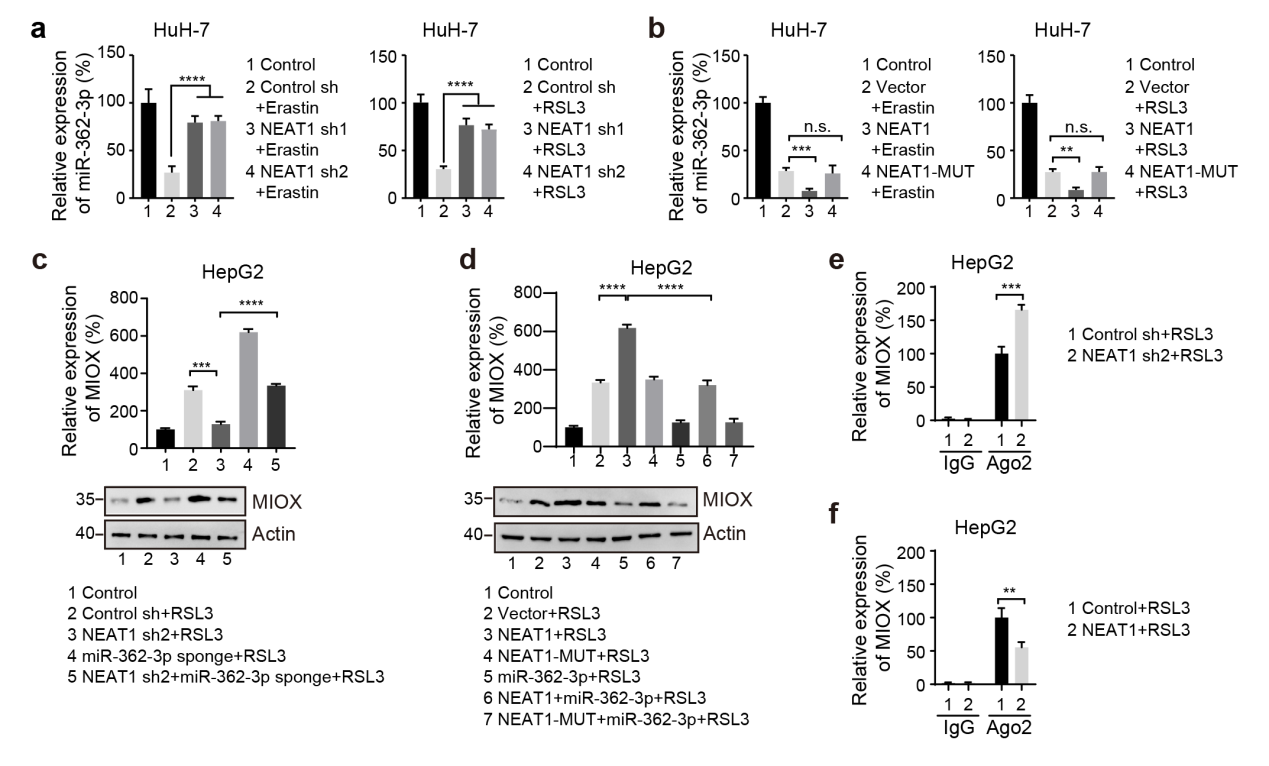


**Supplementary Fig. 5 NEAT1 promotes the expression of MIOX by regulating miR-362-3p. a, b** Knockdown of NEAT1 promoted miR-362-3p expression in ferroptosis (**a**), whereas overexpression of NEAT1, but not the mutant form, suppressed miR-362-3p (**b**). HuH-7 cells transfected with indicated constructs were treated with erastin (5 µM) or RSL3 (0.5 µM) for 12 h, and the mRNA level of miR-362-3p was measured by qRT-PCR. Control group represents non-transfected and non-treated cells. **c, d** Knockdown of miR-362-3p increased the expression of MIOX inhibited by NEAT1 shRNA (**c**), whereas overexpression of miR-362-3p decreased the expression of MIOX promoted by NEAT1 (**d**). HepG2 cells transfected with indicated constructs were treated with RSL3 (0.5 µM) for 12 h. The expression of MIOX was detected by qRT-PCR and western blot. See Supplementary figure 8 for uncropped western blot image. Control group represents non-transfected and non-treated cells. **e, f** The interaction between MIOX and miR-362-3p was affected by the expression level of NEAT1, which was verified by RIP assay using anti-Ago2 antibody, followed by qPCR to detected the MIOX level. Data shown represent mean ± SD from three independent experiments. ***p* < 0.01; ****p* < 0.001; *****p* < 0.0001; n.s., not significant.


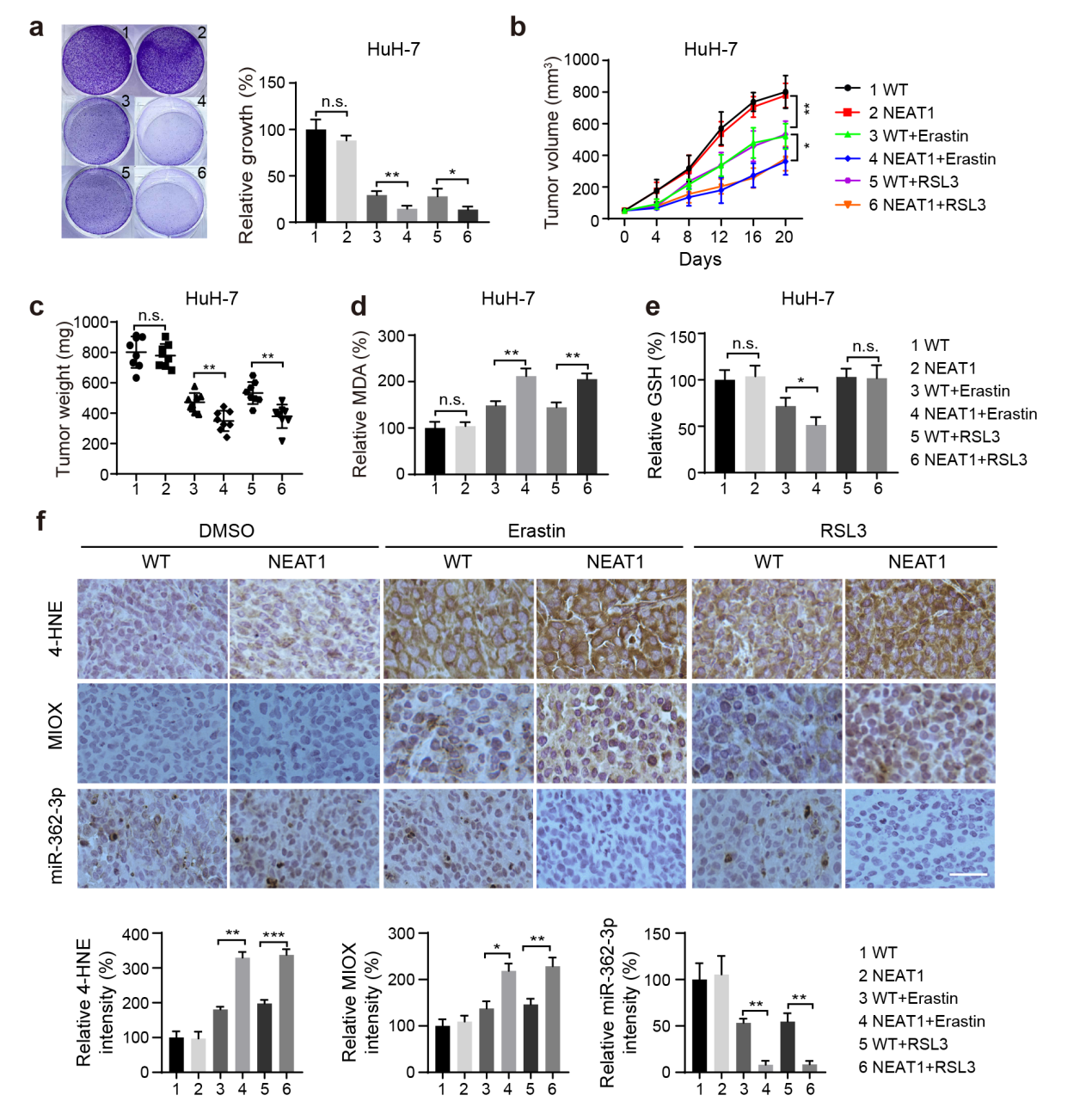


**Supplementary Fig. 6 NEAT1 enhances erastin- and RSL3-induced ferroptosis *in vitro* and *in vivo*.** **a** Overexpression of NEAT1 promoted erastin- and RSL3-induced ferroptosis detected by the colony formation assay. HuH-7 cells were treated with DMSO, erastin (5 µM), or RSL3 (0.5 µM) for 12 h and cultured for 10 days without erastin or RSL3, and the number of cell colonies was calculated. **b-e** Overexpression of NEAT1 enhanced erastin- and RSL3-induced ferroptosis *in vivo*. Corresponding HuH-7 cells were cultured and injected subcutaneously into 7-week-old immunodeficient mice (8 mice per group) at 5 × 10^6^ cells per mouse, and erastin (15 mg/kg, twice every other day) or RSL3 (10 mg/kg, twice every other day) were injected intraperitoneally when the tumor volume of the mice reached 50 mm^3^. Tumor volume (**b**) was measured every 4 days, tumor weight (**c**) was measured on day 20. The relative levels of MDA (**d**) and GSH (**e**) were measured. **f** The expression level of 4NHE, MIOX and miR-362-3p of tumor xenografts was detected by immunohistochemical analysis and in situ hybridization staining. of miR-362-3p. Quantitative analysis of intensity was also shown. Scale bar, 50 µm. The experiment was repeated twice independently with similar results. Data shown represent mean ± SD from three independent experiments. *, *p* < 0.05; **, *p* < 0.01; ***, *p* < 0.001; n.s., not significant.

fig 2b HuH-7 NEAT1_1 (Erastin)

fig 2b HepG2 NEAT1_1 (Erastin)


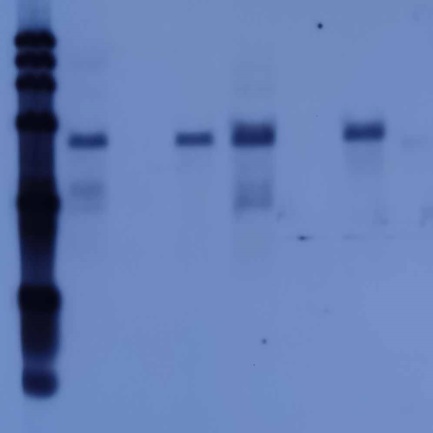

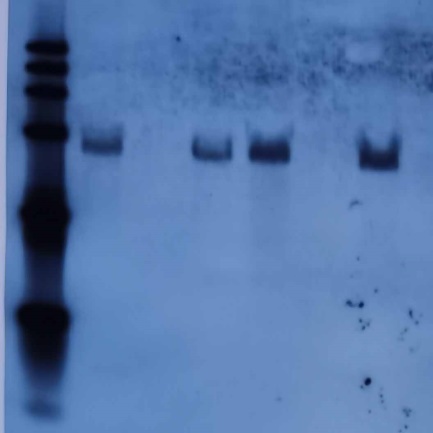


fig 2b HuH-7 NEAT1_2 (Erastin)

fig 2b HepG2 NEAT1_2 (Erastin)


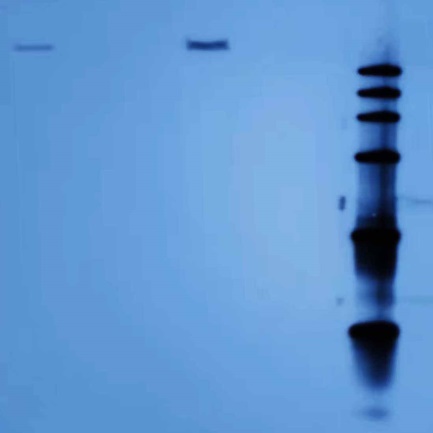

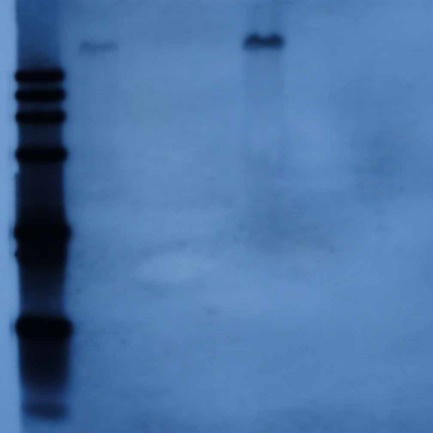


fig 2b HuH-7 GAPDH (Erastin)

fig 2b HepG2 GAPDH (Erastin)


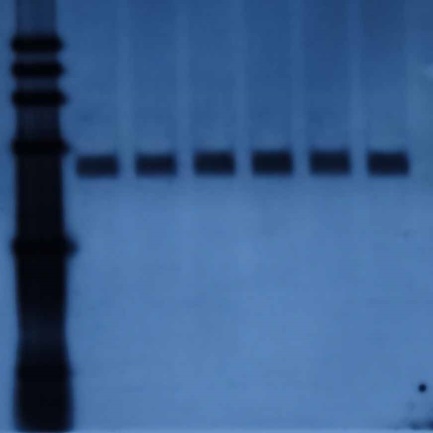

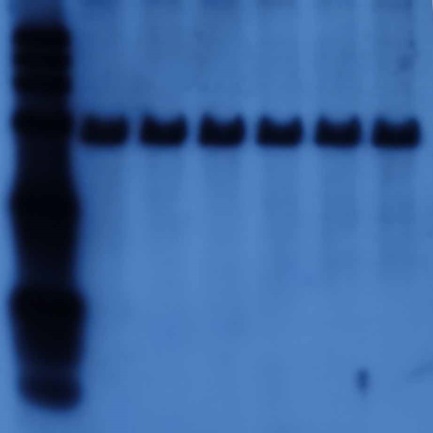


fig 2b HuH-7 NEAT1_1 (RSL3)

fig 2b HepG2 NEAT1_1 (RSL3)


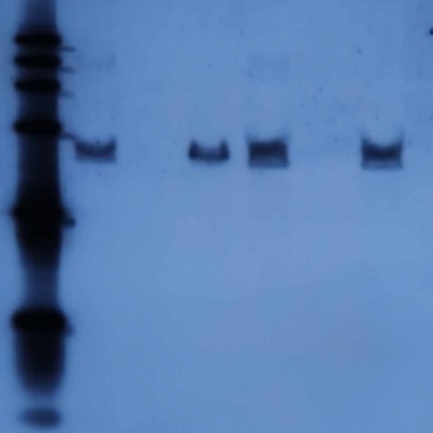


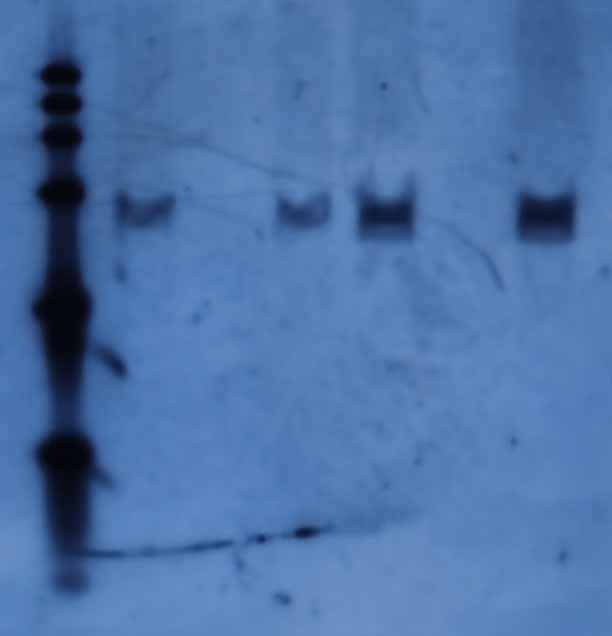


fig 2b HuH-7 NEAT1_2 (RSL3)

fig 2b HepG2 NEAT1_2 (RSL3)


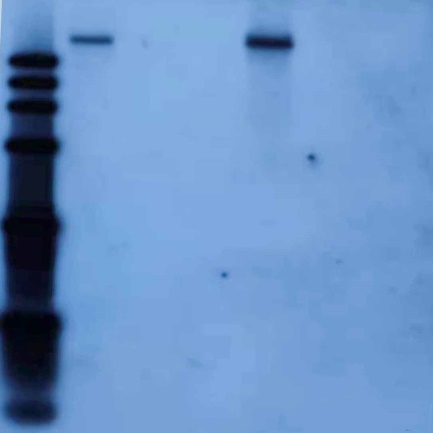

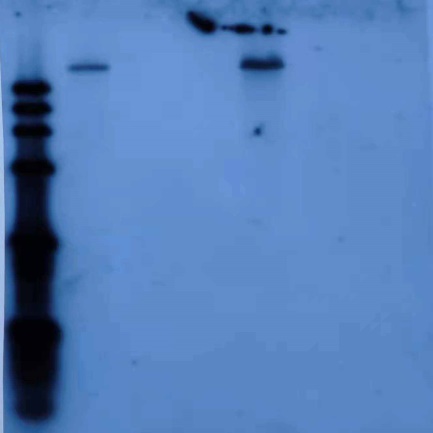


fig 2b HuH-7 GAPDH (RSL3)

fig 2b HepG2 GAPDH (RSL3)


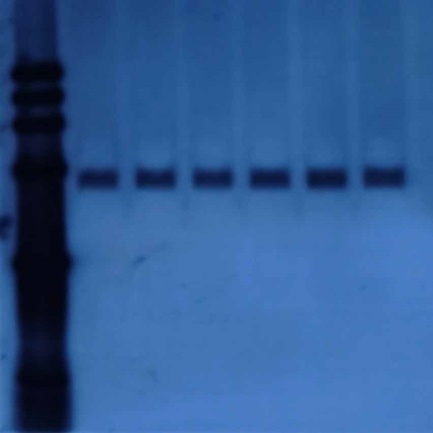

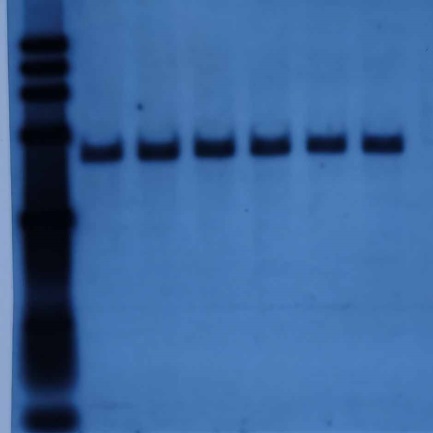


**Supplementary Fig. 7** The uncropped northern blot images.

fig 5j HepG2 Actin

fig 5j HepG2 MIOX


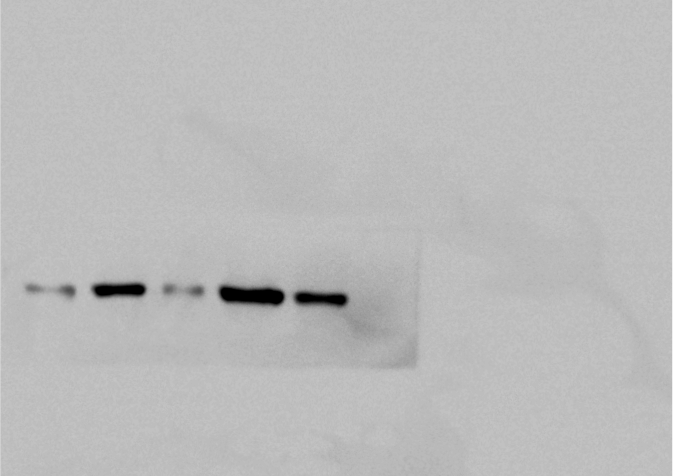




fig 5k HepG2 Actin

fig 5k HepG2 MIOX







fig S2c HepG2 Actin (sh1)

fig S2c HepG2 MIOX (sh1)







fig S2c HuH-7 Actin (sh1)

fig S2c HuH-7 MIOX (sh1)







fig S2c HepG2 Actin (sh2)

fig S2c HepG2 MIOX (sh2)







fig S2c HuH-7 Actin (sh2)

fig S2c HuH-7 MIOX (sh2)







fig S3a HepG2 Actin

fig S3a HepG2 MIOX







fig S3a HuH-7 Actin

fig S3a HuH-7 MIOX







fig S5c HepG2 Actin

fig S5c HepG2 MIOX







fig S5d HepG2 Actin

fig S5d HepG2 MIOX







**Supplementary Fig. 8** The uncropped western blot images.
